# Impact of sex on humoral immunity with live influenza B virus vaccines in mice

**DOI:** 10.1038/s41541-024-00827-x

**Published:** 2024-02-26

**Authors:** Stivalis Cardenas-Garcia, C. Joaquín Cáceres, Aarti Jain, Ginger Geiger, Jong-Suk Mo, L. Claire Gay, Brittany Seibert, Algimantas Jasinskas, Rie Nakajima, Daniela S. Rajao, D. Huw Davies, Daniel R. Perez

**Affiliations:** 1 Department of Population Health, College of Veterinary Medicine, University of Georgia, Athens, GA 30602 USA; 2 Department of Physiology and Biophysics, School of Medicine, University of California Irvine, Irvine, CA 92697 USA

**Keywords:** Infection, Recombinant vaccine

## Abstract

Influenza B virus (FLUBV) poses a significant infectious threat, with frequent vaccine mismatch limiting its effectiveness. Our previous work investigated the safety and efficacy of modified live attenuated FLUBV vaccines with rearranged genomes (FluB-RAM and FluB-RANS) or a temperature-sensitive PB1 segment with a C-terminal HA tag (FluB-att). In this study, we compared the immune responses of female and male DBA/2J mice vaccinated with these vaccines, including versions containing a chimeric HA segment with an N-terminal IgA-inducing peptide (IGIP). Importantly, both recombinant viruses with and without IGIP remained genetically stable during egg passage. We found that introducing IGIP strengthened vaccine attenuation, particularly for FluB-RAM/IGIP. Prime-boost vaccination completely protected mice against lethal challenge with a homologous FLUBV strain. Notably, recombinant viruses induced robust neutralizing antibody responses (hemagglutination inhibition titers ≥40) alongside antibodies against NA and NP. Interestingly, female mice displayed a consistent trend of enhanced humoral and cross-reactive IgG and IgA responses against HA, NA, and NP compared to male counterparts, regardless of the vaccine used. However, the presence of IGIP generally led to lower anti-HA responses but higher anti-NA and anti-NP responses, particularly of the IgA isotype. These trends were further reflected in mucosal and serological responses two weeks after challenge, with clear distinctions based on sex, vaccine backbone, and IGIP inclusion. These findings hold significant promise for advancing the development of universal influenza vaccines.

## Introduction

The incidence of FLUBV infections varies from season to season worldwide. During the 2018–2019 and the 2019–2020 influenza seasons, FLUBV showed an early onset and increasing trend compared to previous seasons^1–4^. This trend was halted by the emergence of the SARS-CoV-2 pandemic virus that forced social distance, self-and/or government-imposed quarantines among other prevention approaches leading to a drastic reduction in the burden of influenza and other respiratory diseases^1–4^.

Vaccination is the primary defense against influenza virus infections^5^. In the US, the list of FDA-approved influenza vaccines includes inactivated split-virion influenza virus vaccines (IIV), recombinant HA proteins produced in baculovirus expression systems (rIV), and live-attenuated influenza vaccines (LAIV)^6^. These vaccines are presented as trivalent or quadrivalent formulations. Trivalent formulations are composed of two influenza A virus (FLUAV) strains representing the H1N1 and H3N2 subtypes, and only one FLUBV strain from either the Victoria lineage or the antigenically distinct Yamagata lineage. Quadrivalent formulations include both FLUBV lineage strains. Despite continuous seasonal vaccine updates, vaccine mismatch occurs frequently for FLUAV and FLUBV. FLUBV vaccine mismatch is particularly common in areas of the world where trivalent vaccine formulations are in use^7–14^. Of note, quadrivalent LAIV formulations introduced since the 2013–2014 influenza season have been associated with low efficacy in protecting against seasonal influenza viruses, particularly in children^6^ underscoring the need for further vaccine research aimed at developing more effective and broadly protective vaccines^15^.

Previous studies have shown that intranasal administration of LAIVs provides broader humoral and cellular immune responses compared to vaccines administered parenterally, likely due to the former establishing virus replication that mimics a natural infection^16^. In the US, the FDA-approved LAIVs are based on cold-adapted (ca) master donor virus backbones developed in the 1960s, ca-A/Ann Arbor/6/1960 and ca-B/Ann Arbor/1/1966. We have previously developed a series of alternative attenuation strategies for both FLUAV and FLUBV strains based on a combination of either genome rearrangement or temperature-sensitive mutations with the addition of epitope tags^17–20^. These strategies have been proven safe and effective in pre-clinical studies. For FLUBV LAIVs, we generated FluB-RAM (rearranged PB1 and M segments), FluB-RANS (rearranged PB1 and NS segments) and FluB-att (PB1 with temperature sensitive mutations and C-terminal HA tag). Unlike current LAIVs, these platforms carry the inherent potential to update the entire vaccine virus backbones, not just the surface gene segments.

The addition of adjuvants to the vaccine formulation is known to increase the strength of the immune response^21^, and immunomodulators to the vaccine can have similar effects. The IgA inducing protein (IGIP) is an immunomodulator initially identified in the bovine gastrointestinal-associated lymphoid tissue (GALT). IGIP is highly conserved among mammals and a very small protein with a predicted molecular weight for the entire peptide between ~5.1 and ~5.9 KDa^22^. IGIP is secreted by antigen-presenting dendritic cells in the intestinal tract, and it was shown to positively regulate mucosal IgA expression in the gut^22,23^. Mucosal IgA is one of the first lines of defense and plays an important role in protection against influenza viruses, with the ability to induce cross-protection against multiple influenza subtypes^24–27^. We previously showed that incorporation of IGIP into the genome of prototypical FLUAVs of human and swine-origin improves virus attenuation and can potentially enhance mucosal IgA and IgG responses^18^. We hypothesize that the addition of IGIP to the FLUBV LAIV vaccine candidates^17^ would improve virus attenuation without compromising protection and antibody response stimulation. We previously observed that male DBA/2J mice showed greater susceptibility to FLUBV than female mice^17^. Therefore, the main objective of this study was to perform systematic analyses to understand the influence of IGIP in the humoral immune responses after vaccination with FLUBV LAIVs and how biological sex modulates such responses. Thus, the IGIP was incorporated into 3 different FLUBV LAIV platforms (FluB-RAM/IGIP, FluB-RANS/IGIP, and FluB-att/IGIP), and their safety, immunogenicity, and protection were analyzed in male and female mice challenged with a lethal dose of a homologous FLUBV strain.

## Results

### FLUBV chimeric IGIP-HA segment and stability of recombinant viruses

**Fig. 1:**
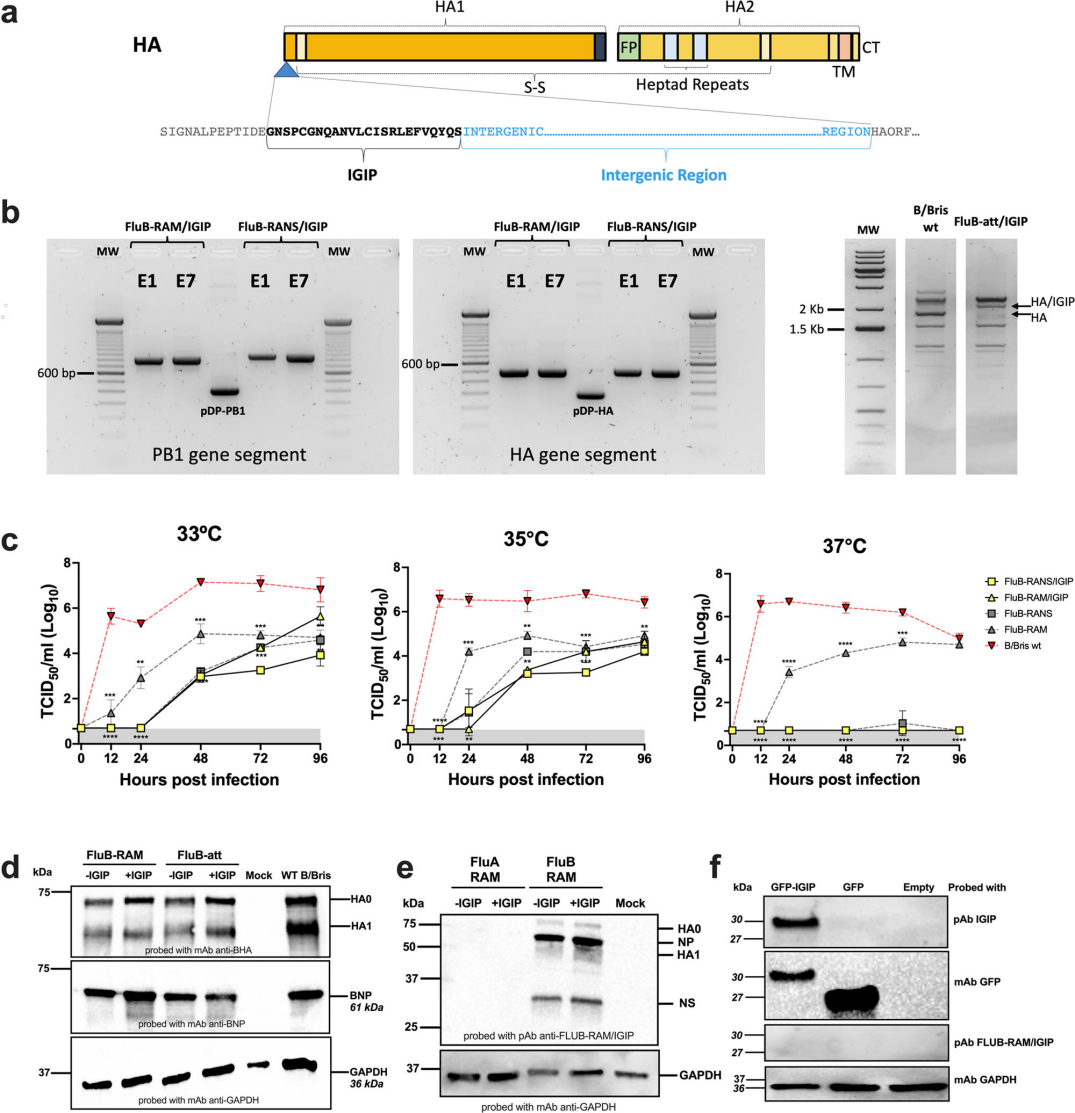
Design, stability, and growth kinetics of FLUBV recombinants carrying IGIP. **a** Schematic of chimeric IGIP-HA segment 4 of FLUBV. The IGIP sequence is cloned downstream the HA signal peptide sequence and upstream of peptide sequences that allow the release of IGIP from the mature HA protein. **b** Gene segment stability. Rearranged PB1 (left panel) and IGIP-HA (middle panel) in FluB-RAM/IGIP and FluB-RANS/IGIP analyzed by RT-PCR with specific primers (sequences available upon request). Differences in migration patterns of E1 and E7 PCR products and corresponding non-IGIP plasmids used as controls. MS-RT-PCR (right panel) of non-IGIP FLUBV strain control and FluB-att/IGIP strain. Migration pattern of HA between the two strains is shown and compared to a molecular weight marker ladder on the left. **c** Growth kinetics analyses at different temperatures of recombinant FLUBV constructs and FLUBV wild type strain. Yellow symbols correspond to FluB-RAM/IGIP (triangle) and FluB-RANS/IGIP (squares). The FLUBV wild type (FLUBV wt, red symbols) and non-IGIP FLUBV (gray symbols) growth kinetics curves were previously reported and shown in this graph for comparison since those were performed simultaneously^18,29^. Virus growth kinetics were analyzed using the Gompertz growth non-linear regression model. Differences in growth rate between viruses were calculated by AUC analysis, followed by Brown–Forsythe and Welch ANOVA plus Dunnett’s T3 post hock analysis. Two-way ANOVA was employed to determine virus growth differences by timepoint. Statistically significant differences between viruses’ growth rates are denoted by different letters. **d** Detection of BHA and BNP expression in vitro via Western blot. MDCK cells were infected with FluB-RAM, FluB-RAM/IGIP, FluB-att, FluB-att/IGIP, and B/Bris WT. Protein extracts were used to detect FLUBV-HA and FLUBV-NP. As a loading control, GADPH was detected (additional details in Supplementary Fig. 1). **e**, **f** Absence of anti-IGIP antibodies after vaccination with FluB-RAM/IGIP. **e** Protein extracts of cells infected with FLUAV viruses (with and without IGIP), FluB-RAM, and FluB-RAM/IGIP were analyzed by Western blot using polyclonal antibody (pAb) from mice vaccinated with FluB-RAM/IGIP. GADPH was detected as a loading control (additional details in Supplementary Fig. 2). **f** HEK293T cells were transfected with 10 µg of pCAGGS GFP-IGIP, pCAGGS GFP, or pCAGGS empty. A mAb anti-IGIP, mAb anti-GFP, or pAb FluB-RAM/IGIP was used. GADPH was detected as a loading control (additional details in Supplementary Fig. 3). Numbers on the right in **d**–**f** indicate molecular weights from molecular weight markers. The predicted molecular weights of target antigens are denoted in italics.

We previously demonstrated the incorporation of IgA inducing protein (IGIP) coding sequence N-terminal to the mature HA ORF of H1 and H3 subtype influenza A viruses (FLUAV). IGIP-H1 and IGIP-H3 HA segments can be stably maintained in recombinant FLUAVs and have the potential to enhance mucosal IgA and IgG responses^18^. A similar strategy was utilized to generate a chimeric IGIP-HA of FLUBV based on the B/Brisbane/60/2008 HA segment (Victoria lineage) (Fig. 1a). The IGIP coding sequence was inserted between the HA signal peptide and the remaining HA coding region. The resulting IGIP-HA FLUBV segment was used in reverse genetics along three different FLUBV backbones previously described^17,20^. The following recombinants viruses were successfully rescued and propagated in ECEs: FluB-RAM/IGIP, FluB-RANS/IGIP, and FluB-att/IGIP.

Since the recombinant viruses FluB-RAM/IGIP and FluB-RANS/IGIP had the most drastic genomic changes, we assessed the stability of such modifications introduced in the PB1, HA, M, and NS gene segments (Fig. 1b). A similar stability evaluation for the FluB-att/IGIP virus was not performed since we have previously established the genomic stability of the Flu-att backbone^20^. The FluB-RAM/IGIP and FluB-RANS/IGIP viruses underwent six serial passages in ECEs, starting from an E1 virus stock. Segments 1, 4, and 7 from the FluB-RAM/IGIP virus, and segments 1, 4, and 8 from the FluB-RANS/IGIP virus were amplified by RT-PCR and sequenced by Sanger using the E1 and E6 stocks. Results from the targeted RT-PCR, showed the presence of the re-arranged PB1 segments with the expected size changes (820 and 862 base pairs, respectively vs 418 base pairs for the WT PB1), and the modified HA gene (493 base pairs vs 268 base pairs for the WT HA) after serial passages (Fig. 1b). Sequencing analysis confirmed the presence of the BM2 and BNS2 downstream of the PB1 gene segment in FluB-RAM/IGIP and FluB-RANS/IGIP, respectively. Mutations engineered to prevent expression of BM2 from segment 7 in FluB-RAM/IGIP or NS2 from segment 8 in FluB-RANS/IGIP were maintained in E6 as designed. Integrity of the IGIP sequence as well as additional modifications in the HA segment from FluB-RAM/IGIP and FluB-RANS/IGIP were confirmed after the serial passages. Multi-segment RT-PCR was performed using RNA extracted from the FluB-att/IGIP stock (Fig. 1b). The HA gene shows the expected size shift when compared to that of a non-IGIP FLUBV. Sequencing analysis also confirmed the integrity of PB1 mutations introduced in the FluB-att/IGIP virus. The stability of IGIP-coding viruses resembles the one from the non-IGIP counterparts^17,20,28^.

### Recombinant FLUBVs carrying IGIP-HA segments display temperature-dependent restrictive growth in vitro

Growth kinetic analysis was performed in MDCK cells infected with MOI of 0.01 and incubated at 33°, 35°, and 37 °C (Fig. 1c). Compared to the B/Bris WT virus, and the non-IGIP FluB rearranged controls, the FluB-RAM/IGIP and FluB-RANS/IGIP showed significantly lower replication at all three temperatures, as supported by the AUC, Brown-Forsythe and Welch ANOVA, and Two-way ANOVA analyses. At 37 °C, titers of both IGIP viruses were under the limit of detection. In contrast, the non-IGIP FluB-RAM virus still maintained significant replication at 37 °C although about 2 log_10_ lower than the B/Bris WT virus control (between 12– 96 hpi)^17^. Although we did not test whether the FluB-att/IGIP had a similar temperature sensitive phenotype, previous data with FluB-att and the data in vivo described below suggest an attenuated profile.

### The incorporation of IGIP does not impact HA expression in vitro

MDCK cells were infected with FluB-RAM, FluB-RAM/IGIP, FluB-att, FluB-att/IGIP and B/Bris WT at 35 °C. At 16 h post-infection, protein lysates from virus-infected cells were analyzed by Western blot to detect expression of BHA and BNP. The housekeeping protein GADPH was used a control of protein loading in Western blots (Fig. 1d). The results show that incorporation of IGIP does not appear to impact the expression and processing of HA regardless of virus background. A slightly lower expression of BHA and BNP was observed in cells infected with any of the FLUBV LAIVs compared to the B/Bris WT control, which is consistent with the growth kinetics patterns at 35 °C (Fig. 1c).

### Absence of anti-IGIP antibodies after vaccination with FLUB LAIVs-IGIP

To determine whether vaccination with recombinant viruses encoding IGIP would lead to antibodies against IGIP itself, protein extracts from cells infected with either FluA-RAM, FluA-RAM/IGIP, FluB/RAM- or FluB-RAM/IGIP viruses were probed by Western blot using polyclonal antibodies (pAb) from mice previously vaccinated with the FluB-RAM/IGIP virus (Fig. 1e). Sera from the FluB-RAM/IGIP-vaccinated group was chosen because the virus is the least defective in replication in vitro among the three IGIP recombinant viruses. Antibodies that recognized proteins from FLUBV-infected cell lysates, but not FLUAV-infected controls were readily observed using sera from FluB-RAM/IGIP-vaccinated mice. The protein migration profile of the samples from FluB/RAM- and FluB-RAM/IGIP was similar. The lack of discernible protein bands from the samples of FluA-RAM/IGIP-infected cells suggest a lack of antibodies against IGIP in sera from FluB-RAM/IGIP-vaccinated mice. To confirm this, we transfected HEK293T cells with a pCAGGS vector containing a chimeric GFP-IGIP construct, only GFP or empty pCAGGS as control (Fig. 1f). Western blot analysis revealed detection by the anti-IGIP mAb of a protein band consistent with the predicted migration of GFP-IGIP (~30 kDa). In addition, the detection of IGIP was only possible in the cells transfected with GFP-IGIP, whereas cells transfected with GFP, or empty vector did not show the detection of any protein, confirming the specificity of the anti-IGIP mAb. In contrast, GFP (~27 kDa) was readily detected from cells transfected with pCAGGS GFP-IGIP and pCAGGS GFP. After the expression of IGIP was confirmed, we probed the protein extract with the pAb FluB-RAM/IGIP, confirming the lack of anti-IGIP antibodies.

### Recombinant FLUBVs carrying IGIP-HA segments are attenuated in vivo

**Fig. 2:**
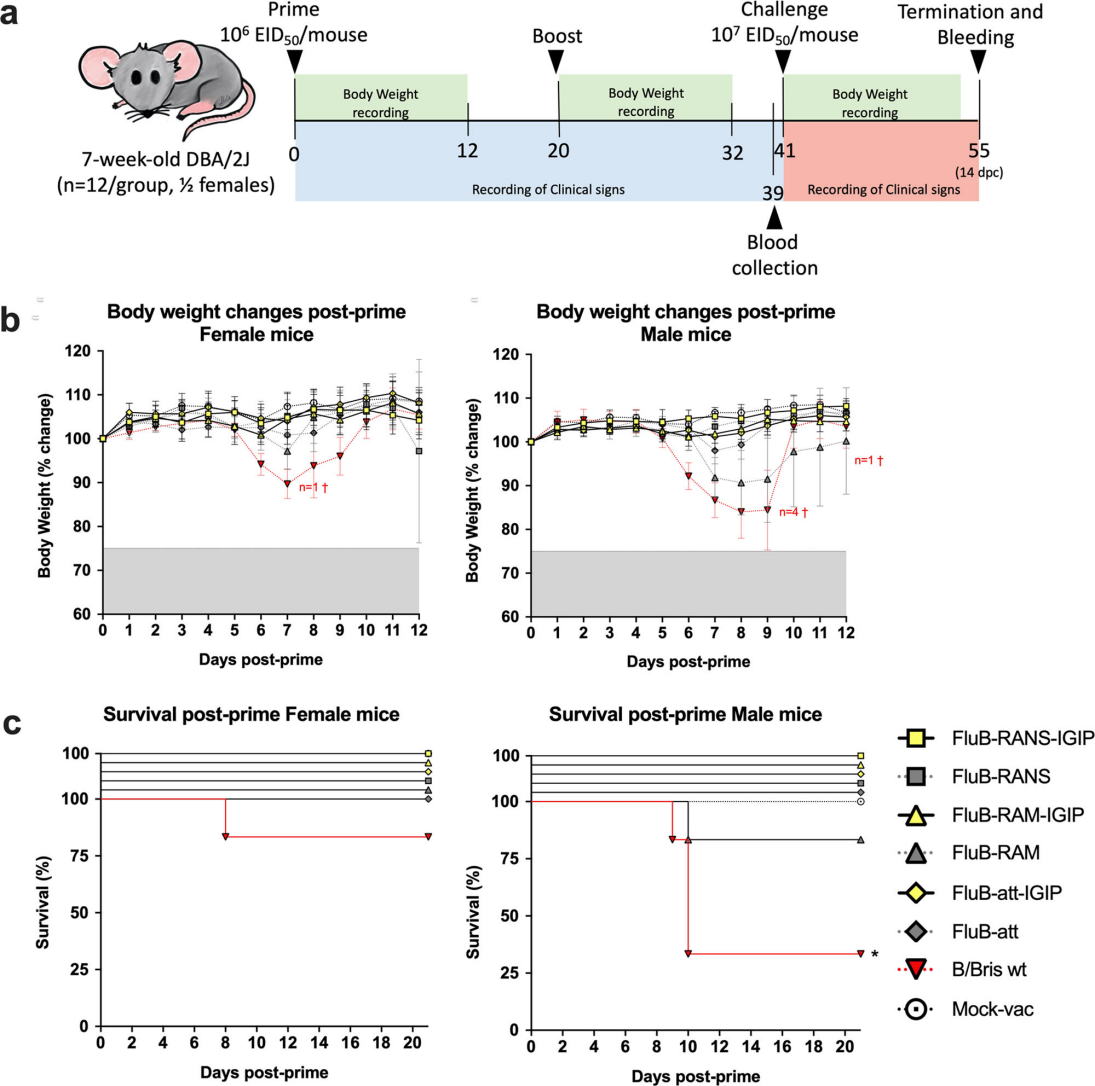
Vaccine study design. **a** Safety and efficacy vaccine study design follows previously reported design^18,29^. Briefly, 7-weeks old DBA/2J mice, ½ female were primed and boosted, 3 weeks apart with one of the recombinant FLUBV vaccines. At 3 weeks post-boost, mice were challenged with an aggressive dose of the B/Bris PB2 F406Y strain. Mice were monitored for clinical signs and survival after prime, boost and challenge as indicated. **b** Body weight changes and **c** survival post-prime virus inoculation of female (left panels) and male (right panels) mice. Yellow symbols correspond to groups of mice inoculated with IGIP-containing FLUBV recombinants. Gray symbols correspond to mice inoculated with non-IGIP FLUBV recombinants whereas red symbols correspond to mice inoculated with the FLUBV wt strain (as previously reported^18,29^). Number of mice that succumbed to virus inoculation are denoted with the “†” symbol with colors matching the corresponding group.

DBA/2J mice are susceptible to FLUBV without further adaptation and a good animal model for vaccine efficacy studies^17,20,29^. Please note that the safety and efficacy profile of the FluB-RAM, FluB-RANS, and Flu-att vaccine candidates has been previously demonstrated^17,20,29^. Since the IGIP-containing recombinant FLUBV vaccines were tested at the same time, some of the previously published data is shown to provide better context on this report. Female and male mice (7-week-old) were inoculated with 10^6^ EID_50_/mouse i.n. following a prime/boost strategy, 20 days apart (Fig. 2a). Mice were inoculated with the corresponding recombinant FLUBV vaccine candidate or B/Bris WT control virus. An additional group of mice were inoculated with PBS as mock vaccine controls. As previously described, male and female mice inoculated with the B/Bris WT showed clinical signs of disease associated with body weight loss leading to 4 male (total *n* = 6) and 1 female (total *n* = 6) mouse to be humanely euthanized (Fig. 2b, c). The recombinant viruses were attenuated compared to the B/Bris WT and showed different levels of attenuation, not only depending on the virus genomic modifications but also on biological sex. After prime, the least attenuated was the FluB-RAM virus followed by the Flu-att virus. Both viruses were noticeably less attenuated in male than female mice. This was particularly the case in male mice inoculated with the FluB-RAM virus in which significant body weight loss was observed, and one mouse (out of *n* = 6) was humanely euthanized compared to female (*n* = 6) mice that showed some body weight loss, but all survived. Incorporation of IGIP led to substantial attenuation of all viruses, with male and female mice showing unremarkable body weight changes and 100% survival as those in the mock vaccinated group. Although formal mouse lethal dose 50 s for each of the FluB-LAIVs was not performed, the data presented suggest being higher than 10^7^ EID50/mouse which positions them as 10 – 100-fold lower than the B/Bris WT strain. Boost vaccination resulted in no clinical signs and 100% survival in all groups (data not shown).

### Influence of sex on humoral response post-boost vaccination

**Fig. 3:**
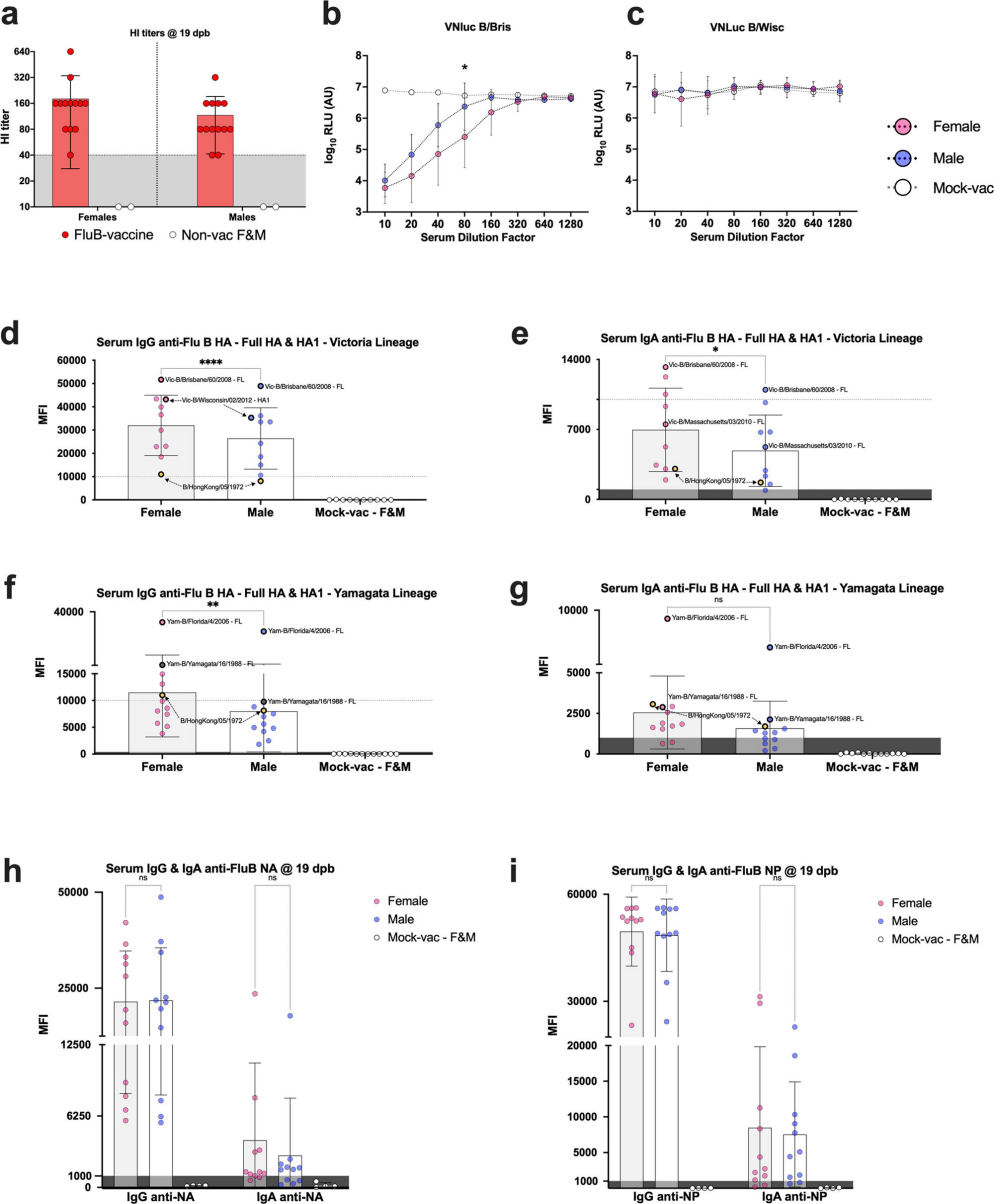
Biological sex vs serological responses to influenza B virus antigens at 19 days post-boost vaccination. **a** Anti-B/Bris HI titers (left panel) segregated by biological sex irrespective of the vaccine received. **b** VNluc titers against homologous B/Bris PB1Nluc (middle and **c**) antigenically unmatched B/Wisc PB1Nluc strains. Nluc activity in relative light units (RLU) is a surrogate for antibody virus neutralization titers. **d**–**i** Serum samples segregated based on biological sex probed to protein microarrays with a panel of FLUBV antigens. Results expressed as the group mean fluorescence intensity (MFI) ± SD. Data points in **d** through **g** show MFI against each HA protein on the array. Some data points are labeled with the corresponding protein and thick borders to show the consistency of relative reactivity of antibodies to those antigens. Yellow data points depict the ancestral B/Hong Kong/5/1972 HA protein used as reference to compare reactivities of Victoria- vs Yamagata lineage antigens. Data points in **h** and **i** correspond to reactivity of each serum sample against a single NA and NP antigen on the array, respectively. MFI values of ≤400 and ≤1000 for IgG and IgA reactivity signals, respectively, are considered (dark) background. 2-way ANOVA followed by Tukey’s or Sidak’s (HI data) multiple comparisons tests was performed to determine differences between groups. VNluc curves were analyzed using multiple t tests. *P* values denoted by asterisks (*) as follows: *p < 0.05, **p < 0.01, ***p < 0.001, ****p < 0.0001, and ns non-significant.

The results above suggested that sex is a variable in the susceptibility to both wild type and recombinant FLUBV infection in DBA/2J mice, with increased susceptibility in male compared to female mice. Therefore, we investigated whether the quality of humoral immune responses was different between female and male mice after prime-boost vaccination and two weeks after challenge with a lethal dose of 10^7^ EID_50_/mouse of the B/Bris/PB2 F406Y homologous strain^17,20^. Sera collected at 19 dpb from a subset of 4 mice/vaccine group (2 males, 2 females, except from the FluB-RAM group that contain samples from 1 male and 2 females) was analyzed by HI assays. HI data was arranged based on sex, independent of the type of vaccine received (Fig. 3a). No statistically significant differences were observed between female and male mice, but both sexes displayed HI titers with protective predictive value (HI titer ≥40). Consistent with the HI data, data from a modified virus neutralization assay based on Nluc activity (VNluc) showed that 19 dpb sera from female (*n* = 15) mice had noticeable improved neutralizing activity against the B/Bris/Nluc strain than similar samples from male (*n* = 14) mice (Fig. 3b), particularly at dilution 1/80 (*p* < 0.05). Moreover, consistent with previous results of poor cross HI responses^20,29^, negligible VNluc activity was observed against the Yamagata lineage B/Wis/Nluc virus (Fig. 3c). To further define potential sex differences in serological responses, IgG and IgA antibodies were analyzed using protein microarray reactivity data of 20 HA FLUBV proteins (Table 1) corresponding to the Victoria and Yamagata lineages (Fig. 3d–g), 1 NA protein derived from B/Phuket/3073/2013 (Fig. 3h) and 1 NP protein derived from B/Florida/4/2006 (Fig. 3i). Please note that the data points plotted in Fig. 3d, e represents the average reactivity of each HA antigen on the array probed with sera obtained from female- (*n* = 11) and male- (*n* = 11) vaccinated mice and mock vaccinated (*n* = 4, ½ female). In contrast, data points in Fig. 3h, i represent the reactivity of each serum sample to the single NA and NP antigen on the array. Graphs in subsequent figures follow the same logic for HA, NA, and NP antigens. Serum IgG and IgA reactivity data was consistent with the HI data. Female mice showed statistically higher IgG and IgA levels against Victoria lineage HA antigens (*p* < 0.001 and *p* < 0.05, respectively), and significantly higher anti-Yamagata HA IgG responses (*p* < 0.05) than males (Fig. 3d–f). Although IgA reactivity against Yamagata lineage HA antigens were significantly lower, higher signals were observed from female samples compared to those of male (*p* > 0.05) (Fig. 3g). No statistical significance was observed for the single NA and NP FLUBV proteins analyzed on the array (Fig. 3h, i), particularly of the IgA isotype showing numerically higher average MFI signals in serum samples from female than male mice.

**Table 1:** Protein antigens used in protein microarray analysis

Protein	Region	FLUBV strain	Lineage	Expression system	Catalog no.
HA	HA1	B/Victoria/02/1987	Victoria	HEK293	40163-V08H
HA	HA1	B/Wisconsin/01/2012	Victoria	HEK293	40462-V08H1
HA	HA1	B/Brisbane/60/2008	Victoria	HEK293	40016-V08H1
HA	HA1	B/Ohio/01/2005	Victoria	HEK293	40460-V08H1
HA	HA1	B/Massachusetts/03/2010	Victoria	HEK293	40191-V08H1
HA	HA1	B/Malaysia/2506/2004	Victoria	HEK293	11716-V08H1
HA	HA1	B/Hong Kong/05/1972	Victoria	HEK293	40461-V08H1
HA	HA1	B/Yamagata/16/1988	Yamagata	HEK293	40157-V08H1
HA	HA1	B/Victoria/504/2000	Yamagata	HEK293	40391-V08H
HA	HA1	B/Brisbane/3/2007	Yamagata	HEK293	40431-V08H1
HA	HA1	B/Phuket/3073/2013	Yamagata	HEK293	40498-V08H1
HA	HA1	B/Florida/07/2004	Yamagata	HEK293	40432-V08H1
HA	HA1	B/Utah/02/2012	Yamagata	HEK293	40463-V08H1
HA	HA1	B/Florida/4/2006	Yamagata	HEK293	11053-V08H1
HA	HA1 + HA2	B/Brisbane/60/2008	Victoria	E. coli	40016-V08B
HA	HA1 + HA2	B/Malaysia/2506/2004	Victoria	HEK293	11716-V08H
HA	HA1 + HA2	B/Massachusetts/03/2010	Victoria	Baculovirus	40191-V08B
HA	HA1 + HA2	B/Florida/4/2006	Yamagata	HEK293	11053-V08H
HA	HA1 + HA2	B/Phuket/3073/2013	Yamagata	Baculovirus	40498-V08B
HA	HA1 + HA2	B/Yamagata/16/1988	Yamagata	Baculovirus	40157-V08B
HA	HA1 + HA2	B/Utah/02/2012	Yamagata	Baculovirus	40463-V08B
HA	HA1 + HA2	B/Brisbane/60/2008	Victoria	HEK293	40016-V08H
NA	NA	B/Brisbane/60/2008	Victoria	HEK293	40203-VNAHC
NA	NA	B/Phuket/3073/2013	Yamagata	Baculovirus	40502-V07B
NP	NP	B/Florida/4/2006	Yamagata	Baculovirus	40438-V08B

### Influence of IGIP modification on humoral responses post-boost vaccination and post-challenge

**Fig. 4:**
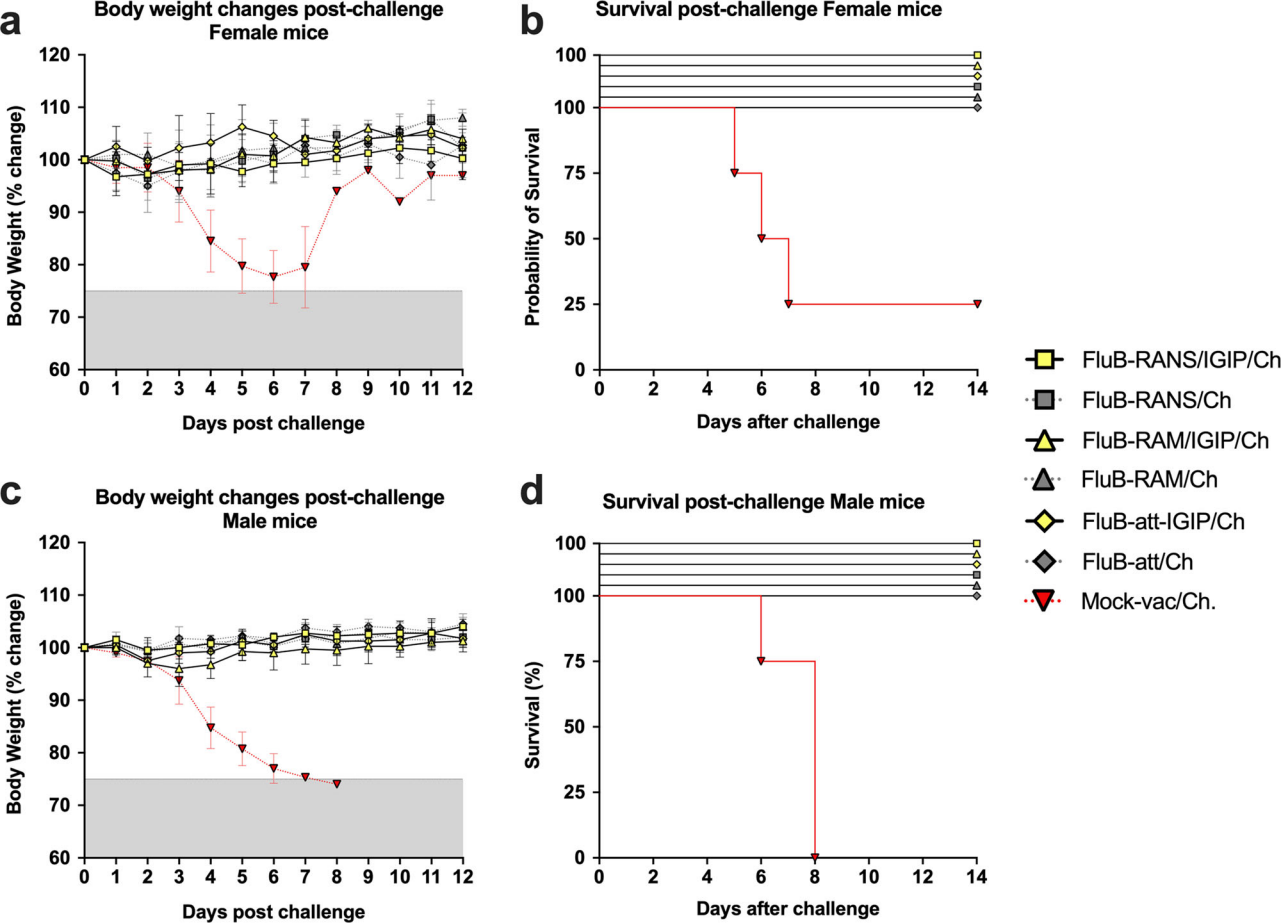
Clinical outcomes post-challenge. **a**–**d** Mice, *n* = 8/group ½ female were challenged with 10^7 EID50/mouse of B/Bris PB2 F406Y virus at 3 weeks post-boost vaccination. Body weight changes and survival were recorded and graphed in Prism 9.3.1 as shown. Yellow symbols correspond to groups of challenged mice previously vaccinated with IGIP-containing FLUBV recombinants. Gray symbols correspond to challenged mice previously vaccinated with non-IGIP FLUBV recombinants whereas red symbols correspond to challenged mice previously mock-vaccinated. All IGIP and non-IGIP FLUBV vaccinated mice show complete protection, 100% survival and negligible clinical signs regardless of biological sex, unlike the mock-vaccinated challenged group.

**Fig. 5:**
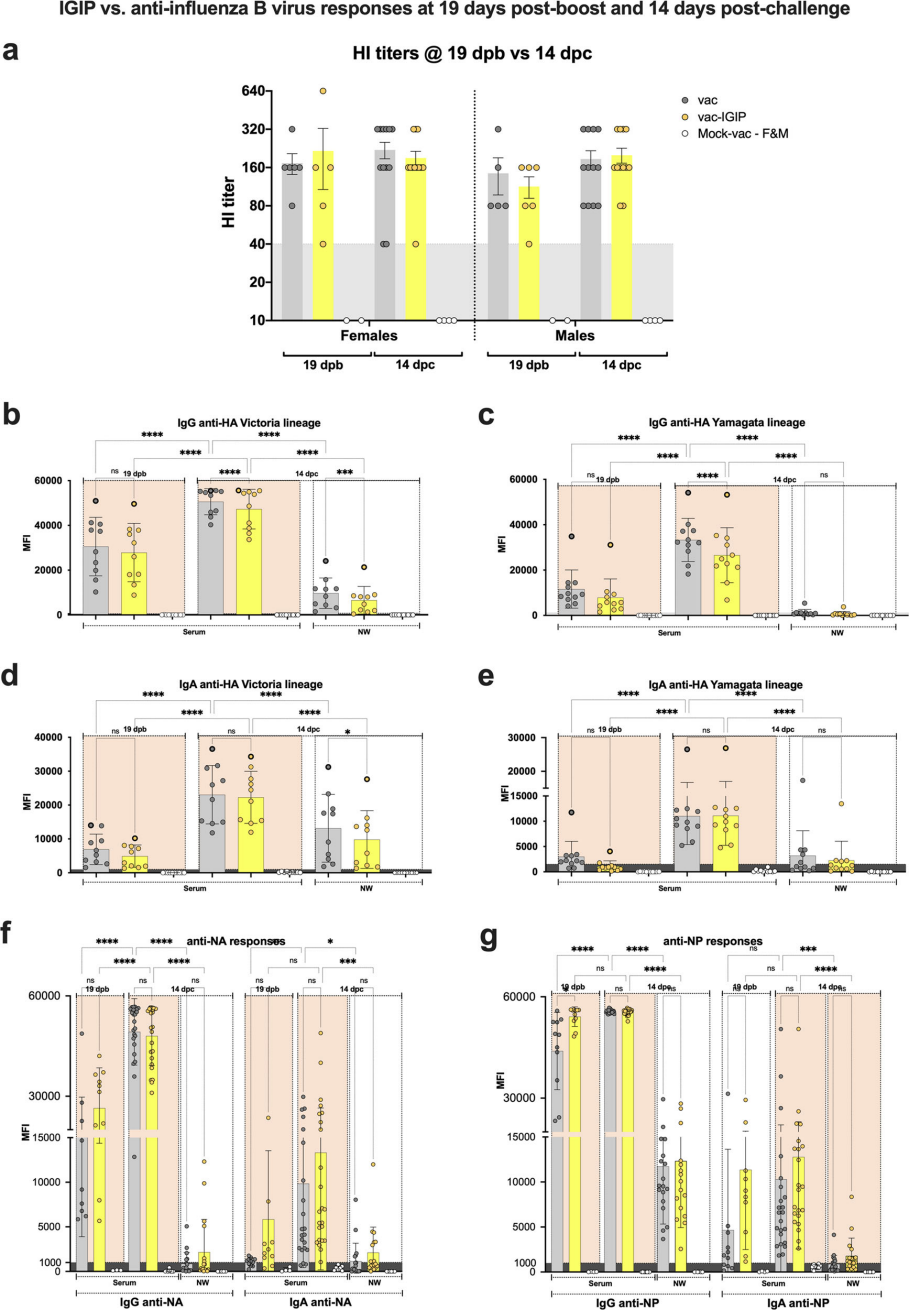
IGIP vs. non-IGIP vaccine groups and effects on anti-influenza B virus responses at 19 dpb and 14 dpc. Serum samples from mice obtained at 19 dpb and 14 dpc were analyzed based on the absence (vac) or presence of IGIP (vac-IGIP) in the FLUBV recombinant vaccine. Except for the HI data (**a**), vac vs vac-IGIP analyses did not discriminate samples based on biological sex. **b**–**g** MFI signals from protein microarray data and statistical analyses follow the same rationale as described in the main text. Statistically significant (asterisks) and non-statistically significant (ns) differences are shown. Statistical analyses were performed using 2-way ANOVA followed by Tukey’s and/or Sidak’s multiple comparisons tests. Statistically significant differences are denoted by asterisks (*) as follows: *p < 0.05: **p < 0.01: ***p < 0.001: ****p < 0.0001: and ns, *p* ≥ 0.05.

Regardless of vaccine virus modifications, all vaccinated mice were fully protected (*n* = 8/vaccine group, ½ female) from lethal B/Bris PB2 406Y virus challenge (Fig. 4a–d). In contrast, all male mice (*n* = 4) and 3 out 4 female mice succumbed to the infection in the mock-vaccinated/challenge group (data previously reported^17^). To better understand the potential role of the IGIP modification on humoral responses, we compared responses between mice vaccinated with the IGIP-encoding vaccines (all combined) and those that received the non-IGIP vaccines (all combined), regardless of sex (Fig. 5a–g). HI data from 19 dpb sera (Fig. 5a) revealed a trend towards lower HI titers in the IGIP group compared to the non-IGIP group in both female and male mice. Similarly, HI data from 14 dpc sera showed a slight increase in HI titers. As with HI titers, the same trend was observed in protein microarray data of IgG and IgA responses against HA antigens of the Victoria and Yamagata lineage in serum samples from 19 dpb and 14 dpc and nasal wash samples from 14 dpc (Fig. 5b–e), where sera and/or NW from non-IGIP vaccinated mice showed significantly higher anti-Victoria lineage responses than those from IGIP vaccinated mice (*p* < 0.0001, for each comparison) (Fig. 5c, d). No significant differences were observed with mice that received the IGIP-encoding vaccine in the responses to NA and NP, except for 19 dpb serum anti-NP IgG (*p* < 0.05). Serum IgG and IgA responses against both Victoria and Yamagata lineage HA protein antigens were significantly lower at 19 dpb vs 14 dpc (*p* < 0.0001, for each comparison). Additionally, 14 dpc serum IgG and IgA responses were significantly higher than those in the NW (*p* < 0.0001, for each comparison) (Fig. 5b–e). Similar effects were observed for anti-NA and NP responses (Fig. 5f, g).

### Influence of vaccine backbone modifications on recall antibody responses based on sex

**Fig. 6:**
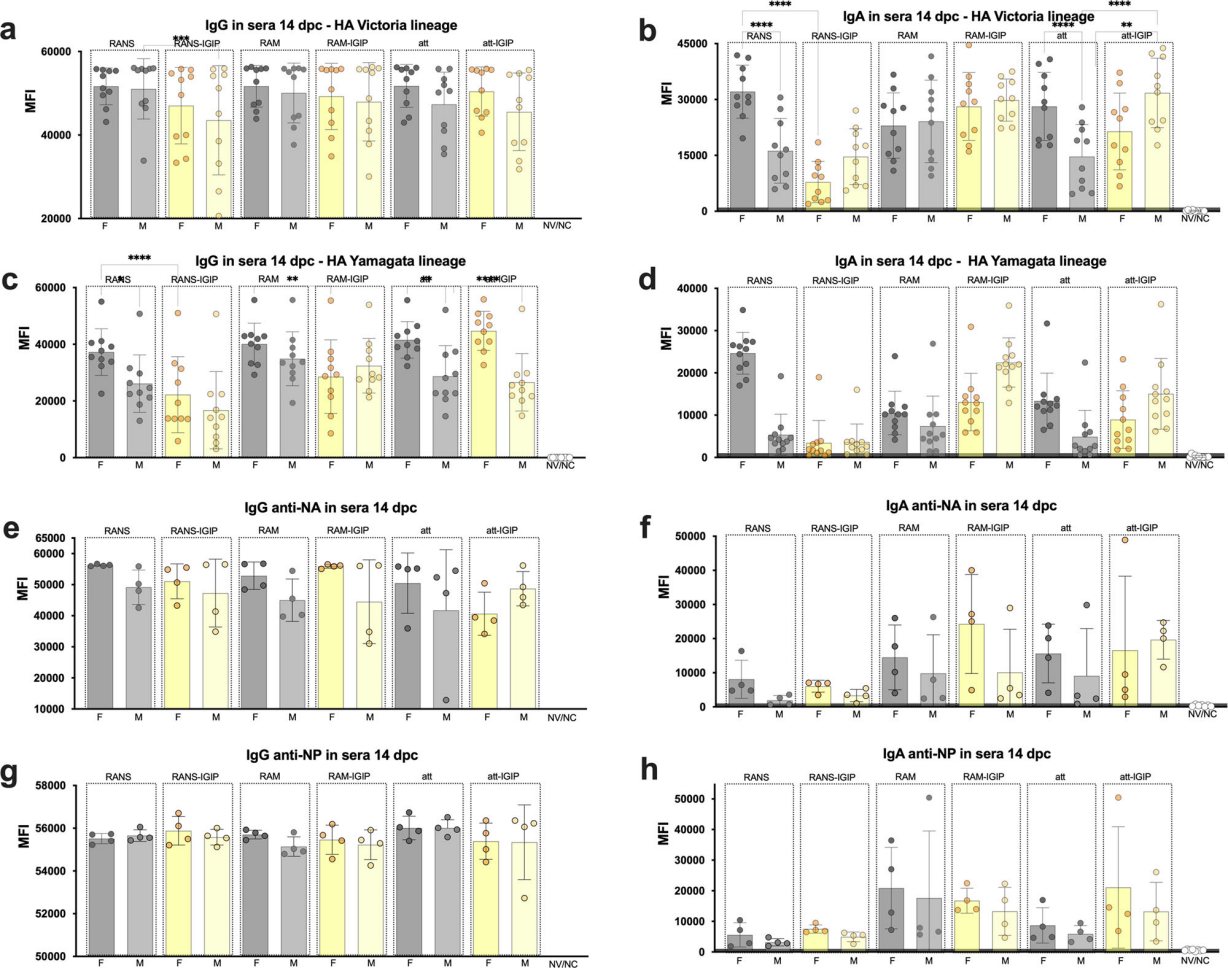
Serological IgG and IgA responses at 14 dpc plotted based on biological sex and vaccine type. **a**–**h** Protein microarray reactivity data measured by MFI as described in Fig. 3. Qualitative differences are observed among different groups based on sex and vaccine type as shown by bar graphs. Statistical analyses included 2-way ANOVA followed by Tukey’s multiple comparison tests (HA antigens) and/or Brown–Forsythe and Welch ANOVA followed by Dunnett’s T3 multiple comparison tests (NA and NP antigens). Biological sex differences and/or IGIP vs non-IGIP differences for each vaccine backbone with statistical significance are noted with asterisks (*) as follows: *p < 0.05: **p < 0.01: ***p < 0.001: ****p < 0.0001: and ns, *p* ≥ 0.05.

**Fig. 7:**
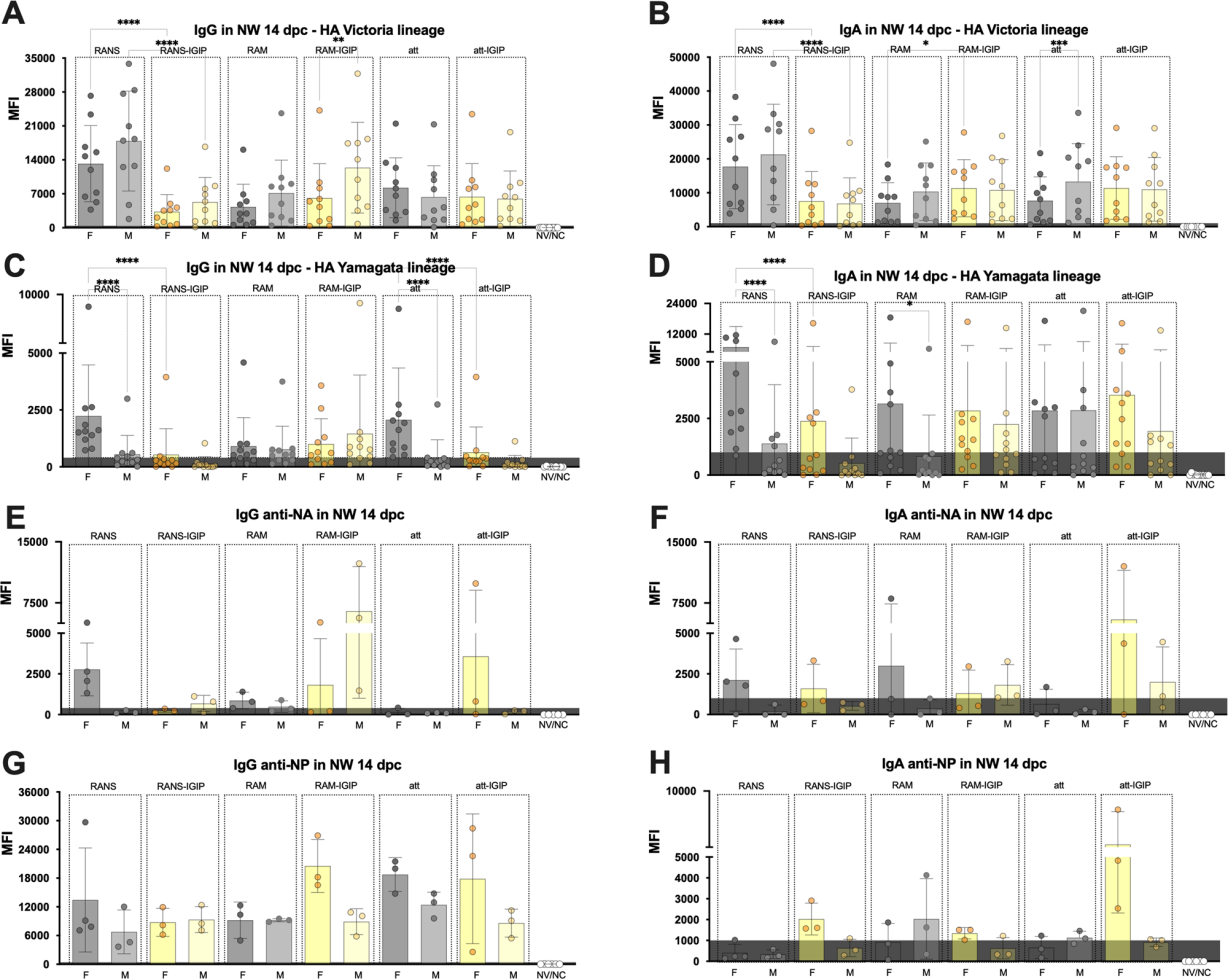
IgG and IgA in nasal washes at 14 dpc plotted based on biological sex and vaccine type. **A**–**H** Protein microarray reactivity data measured by MFI as described in Fig. 3. Statistical analyses included 2-way ANOVA followed by Tukey’s multiple comparison tests (HA antigens) and/or Brown–Forsythe and Welch ANOVA followed by Dunnett’s T3 multiple comparison tests (NA and NP antigens). Qualitative differences shown by bar graphs and statistical differences noted with asterisks (*) as follows: *p < 0.05, **p < 0.01, ***p < 0.001, ****p < 0.0001, and ns, *p* ≥ 0.05.

To further dissect the contributions of the 6 vaccine candidates and the effect of sex on recall antibody responses, the microarray data was analyzed and plotted based on antibody reactivity to each protein antigen in the array, grouped by sex and by vaccine group. Two sets of samples were analyzed, sera and nasal wash samples collected at 14 dpc (Figs. 6 and 7, respectively). With few exceptions as discussed below, consistently higher MFI signals were detected for serum IgG in female (*n* = 4/vaccine group) versus male (*n* = 4/vaccine group) mice against both Victoria and Yamagata HA antigens, however few showed statistically significant differences (Fig. 6a, male mice vaccinated with FluB-RANS>FluB-RANS/IGIP, *p* = 0.002 and 6c, females >males in FluB-RANS, FluB-att and FluB-att/IGIP groups, *p* < 0.05, *p* = 0.001 and *p* < 0.0001, respectively; females in FluB-RANS and FluB-RAM >females in FluB-RANS/IGIP and FluB-RAM/IGIP, *p* < 0.0001 and *p* = 0.007, respectively). No statistically significant differences were observed for either anti-NA serum IgG (Fig. 6e) or anti-NP serum IgG (Fig. 6g) post-challenge although the latter responses are the maximum MFI threshold. IgA responses showed trends to higher signals against Victoria and Yamagata HA antigens in sera from female mice vaccinated with non-IGIP vaccines, while the opposite was observed in sera from male mice vaccinated with the IGIP vaccines (Fig. 6b, d). Statistically significant differences were established for the following comparisons: In Fig. 6b (Victoria lineage HA antigens), females >males in the FluB-RANS and FluB-att groups, *p* < 0.0001; males >females in the FluB-att/IGIP group, *p* = 0.005; females in FluB-RANS group >females in FluB-RANS/IGIP group, *p* < 0.0001 and males in Flu-att/IGIP group >males in FluB-att group, *p* < 0.0001. In Fig. 6d (Yamagata lineage HA antigens), females >males in the FluB-RANS group, *p* < 0.0001; males >females in the FluB-RAM/IGIP group, *p* < 0.05; females in the FluB-RANS group >females in the FluB-RANS/IGIP group, *p* < 0.0001; males in the FluB-RAM/IGIP and FluB-att/IGIP >males in the FluB-RAM and FluB-att groups, respectively, *p* = 0.0004 and *p* < 0.05. Serum IgA responses at 14 dpc against NA and NP remain low, with no statistical differences between females and males. IgG and IgA reactivity in nasal wash samples (Fig. 7) collected at 14 dpc, tended to be higher in male than female samples against homologous Victoria-lineage HAs (Fig. 7a, b), which contrasts with the overall response patterns in serum (Fig. 6a, b). These trends were statistically significant for male>female in the FluB-RAM/IGIP group (IgG, Fig. 7A) and in the FluB-att group (IgA, Fig. 7B). Reactivity in nasal washes, more akin to patterns in serum responses, were observed for the heterologous Yamagata-lineage HAs and the NA and NP antigens with overall increased reactivity displayed by samples from females compared to males (Fig. 7C–H compared to Fig. 6c–h). Statistically significant differences were established for IgG in the FluB-RANS and FluB-att groups (females >males, *p* < 0.0001, Fig. 7C) and among females comparing to groups vaccinated with isogenic vaccines expressing IGIP (FluB-RANS >FluB-RANS/IGIP, FluB-att >FluB-att/IGIP, *p* < 0.0001, Fig. 7C). Additional significant differences were observed for IgA in the FluB-RANS and FluB-RAM groups (females >males) and with females in the FluB-RANS having higher responses than those in the FluB-RANS/IGIP group. No other statistical differences were observed in samples from NW at 14 dpc within each of the vaccine groups, nor between IGIP vs non-IGIP FLUBV constructs (*p* > *0.05*).

## Discussion

We have previously demonstrated that FLUAV LAIV candidates against H1N1 and H3N2 subtypes with HA segments modified to carry the IGIP mature peptide showed improved attenuated profiles compared to the non-IGIP counterparts^18^. This is likely due to IGIP decreasing the fitness of segment 4 (HA). The IGIP was chosen due to its potential as a safe natural vaccine adjuvant that stimulates IgA class switch without side effects^23^. In this report, we expanded the characterization of alternative FLUBV LAIV candidates with similar IGIP modifications on B/Bris HA. The different platforms (FluB-RAM, FluB-RANS, and FluB-att) were used to introduce modified HA segment encoding IGIP resulting in the following vaccine candidates: FluB-RAM/IGIP (rearranged PB1 and M segments), FluB-RANS/IGIP (rearranged PB1 and NS segments) and FluB-att/IGIP (PB1 with temperature-sensitive mutations and C-terminal HA tag). Thus, the vaccines carry multiple modifications that make them less fit than FLUBV WT strains and less likely to reassort at least for the IGIP-HA, PB1-M2/M1 early stops, PB1-NEP/NS1 early stops and/or PB1att gene segments^17,20,29^ depending on the backbone. Studies beyond the scope of this report are needed to determine to which extent the genomic modifications impair the reassortment potential of the modified and non-modified gene segments in the different vaccine backbones with segments from field FLUBV strains.

IGIP-containing FLUBV LAIV candidates showed temperature-sensitive profiles that either resemble or were further impaired compared to the non-IGIP isogenic and already attenuated counterparts. This was most notably the case at 37 °C comparing the FluB-RAM/IGIP versus the FluB-RAM strains (Fig. 1). At 72 hpi and 35 °C, all modified FLUBV strains showed similar titers, ~2 log_10_ lower than the B/Bris WT strain (Fig. 1). More importantly, the IGIP modification was stably maintained over multiple passages in ECEs, as previously realized with the IGIP FLUAV LAIV candidates. Western blot analysis of the HA expression revealed no differences associated with incorporating the N-terminal IGIP (Fig. 1d). This is relevant because it suggests no impairment in neither expression nor processing of HA associated with incorporating IGIP. Further, no reaction was observed between pAb FluB-RAM-IGIP and FLUAV-infected cells, suggesting the lack of anti-IGIP antibodies, whereas pAb FluB-RAM-IGIP recognized proteins from FLUBV-infected cell lysates (Fig. 1e). The migration of protein bands was consistent with the migration of mainly BHA0 and BNS, with a minor band corresponding to the migration of BNP. The latter was confirmed by the lack of reaction between protein extracts of cells transfecting with a plasmid expressing IGIP and the same pAb (Fig. 1f)

The IGIP and non-IGIP FLUBV LAIV candidates reached titers in ECEs that would make them suitable as vaccine candidates (at least 10^8^ EID_50_/mL, except FluB-RANS/IGIP with maximum titer of 10^6^ EID_50_/mL). Incorporation of IGIP in the different FLUBV LAIV candidates also led to improvements of the attenuated profile in vivo. Both, the FluB-RAM/IGIP and FluB-att/IGIP were noticeably more attenuated than the non-IGIP counterparts specially in male mice (Fig. 2b, c).

Previous studies have shown opposite patterns of susceptibility to FLUAV versus FLUBV based on biological sex^17,30,31^. Male mice are more susceptible to FLUBV, but female mice are more susceptible to FLUAV. The differences in susceptibility of male versus female mice infected with FLUBV resemble epidemiological trends in humans where biological males are more prone than biological females to respiratory virus infections^32^. However, influenza risk of hospitalization and mortality depends on several confounding factors (age, pregnancy, co-morbidities) that require more systematic studies to better establish the role of biological sex in influenza disease outcomes^33–36^. We simultaneously tested 6 different FLUBV LAIVs with or without IGIP in 3 different virus backbones. And the use of female and male mice allowed us to explore how these different variables ultimately affected humoral responses before challenge and humoral and mucosal responses after challenge. Serological responses at 19 dpb revealed higher HI, higher VNluc, and higher overall IgG and IgA responses against FLUBV antigens in protein microarrays of samples from female than male mice (Figs. 3, 5, 6, and 7). Sex-dependent and HA lineage-dependent responses were consistent across the different assays that measure anti-HA antibodies (HI, VNluc, and protein arrays). Lack of VNluc inhibitory activity against the Yamagata-lineage B/Wis/Nluc virus with either female or male sera was consistent with the lower IgG and IgA MFI signals in the protein array compared to the same against the Victoria lineage HA antigens. As expected, overall serum IgG MFI signals were higher than those from serum IgA, but both were higher overall in samples from female than male mice. It must be noted that MFI signals depend on several variables besides the relative amounts of probing antibodies. The protein source (mammalian-, baculovirus-, or bacterial-derived), its structural integrity and/or preservation of epitopes of each of the antigens in the array affect its relative reactivity. Therefore, it was important to observe consistency of the relative reactivity of each antigen probed with different sera. The full-length B/Bris HA antigen in the Victoria lineage set provided the highest signal when probed with sera of vaccinated mice. Likewise, the B/Florida/4/2006 full length HA antigen in the Yamagata lineage provided consistently the highest cross-reactive responses.

The incorporation of IGIP on the vaccines had an overall effect of antibody responses that could be explained by two non-mutually exclusive effects. IGIP appeared to reduce responses to HA while enhancing responses to NA and NP. Such observation is consistent with the possibility of IGIP interfering and/or affecting segment 4 transcription/replication and/or HA biogenesis/assembly, which would ultimately favor responses to other viral antigens and perhaps providing a broader response. However, interference alone cannot explain the relatively higher increase in serum IgA responses against NA and NP at 19 dpb. Depending on the vaccine pair comparison, overall higher serum and/or mucosal IgA responses against HA antigens at 14 dpc were observed male mice (FluB-RAM/IGIP vs FluB-RAM) and in male and female mice (FluB-att/IGIP vs FluB-att). It is possible that the potential adjuvant effect of IGIP shows male bias as observed for the IgA responses in groups vaccinated with FluB-RAM/IGIP and FluB-att/IGIP compared to the FluB-RAM and FluB-att groups, respectively (Fig. 7B, D). The data in this report suggest sex-dependent effect of IGIP in which males, more than females, produced mucosal IgA responses better targeted to the homologous Victoria lineage HA antigens. Such pattern was much less obvious in mice that received the non-IGIP vaccines. Interestingly, a previous study showed increased prevalence of mucosal/oral anti-human papillomavirus IgA in men compared to women even though men produce less serum antibodies than women^37^. Analyses of responses at 14 dpc show female bias with trends to higher IgG serum responses and higher IgG and IgA mucosal responses against the heterologous Yamagata lineage HA antigens as well as NA and NP antigens. In the few instances in which these patterns were reversed, those coincided with mice that had received an IGIP-encoding vaccine (serum IgA anti-Yamagata HAs in the FluB-RAM/IGIP and the FluB-att/IGIP groups, Fig. 6d; nasal wash IgG anti-Yamagata HAs and IgG and IgA anti-NA in the FluB-RAM/IGIP group, Fig. 7C, E, F). A female bias with more consistent responses against heterologous Yamagata-lineage HA antigens agrees with the notion of women developing more cross-reactive and/or self-reactive antibodies^38^. It is important to note that serum and NW samples obtained at 14 dpc are from different mice than serum samples obtained at 19 dpb, however biological sex bias and the impact of different FLUBV vaccine modifications were consistently observed for both time points. Additional studies beyond the scope of the present report should consider the host’s genetic background as playing in role in the ability of IGIP to perform as intended. It is well established that in DBA/2J mice re-stimulation of antigen-specific immune responses leads to higher IL-12 responses than Balb/c mice, with the former mouse strain more predisposed to Th1 responses and the latter more predisposed to Th2 responses^39^. In addition, DBA/2J are deficient in CD94, a surface protein primarily expressed by NK cells and a subset of CD8+ T cells and whose role implicates opposite functions as either stimulator or suppressor of immune responses^40^. Overall, our observations are consistent with previous studies assessing the response to vaccination in humans as well as in mice that revealed female bias towards higher antibody responses, higher B cell responses, higher cross-reactive antibodies, and higher CD4+ T cell numbers compared to males^30,31,41–43^. Furthermore, our report strongly suggests that the quality and quantity of immune responses can be influenced by the vaccine platform in both female and male subjects.

Vaccination is considered the first line of defense against influenza. However, significant issues remain with respect to vaccine effectiveness (VE). VE average for the past 16 influenza seasons has been of ~40% (VE range from ~10% for the 2004/05 season to ~60% for the 2010/11 season). VE depends on vaccine strain composition (source: CDC, https://www.cdc.gov/flu/vaccines-work/vaccineeffect.htm) and it decreases with repeated influenza vaccination^44^. Hence, there is an urgent need to revisit the current approach to seasonal influenza vaccination. The SARS-CoV-2 pandemic accelerated the development and approval of highly effective mRNA vaccines. The same mRNA approach for prevention of seasonal influenza is currently being assessed in several clinical trials. It remains to be determined whether mRNA vaccines against influenza will alleviate all or many of the issues faced by current vaccines. In this regard, it is important to note that the IGIP approach could be easily deployed in mRNA vaccine platforms to modulate more effective mucosal immune responses. Alternatively, and if LAIV is the vaccine of choice, our results suggest that the FluB-RAM/IGIP approach provided the least biased IgG and IgA responses between female and male mice against several viral antigens, as observed in samples at 14 dpc. In contrast, the FluB-RANS vaccine results suggest the most asymmetric responses skewed towards higher responses in females than males. Further studies beyond the scope of the present report are required to further dissect potential bias in the immune response in male and female mice vaccinated with different FLUBV LAIV, including those already approved for human use. This is particularly the case to better understand the female/male bias after prime and boost vaccination, which was not possible under the conditions of the experiments presented in this report.

In summary, we showed that different FLUBV LAIV backbones and the incorporation of the chimeric IGIP-HA led to similar protective outcomes but qualitatively different humoral and mucosal response patterns in mice. We chose IGIP in the context of a modified live virus, but its potential adjuvanted effect could be tested in many different vaccine platforms and against multiple diseases. Of note, we further investigated the effect of biological sex on antibody-mediated immune response stimulation. These observations confirmed that female mice are less susceptible to FLUBV than males. However, female mice can mount better and broader antibody responses against FLUBV than males. Further investigation is warranted to broaden our understanding of those factors that drive the opposite-sex-related susceptibilities towards FLUAV and FLUBV.

## Methods

### Ethics statement

Animal studies were approved and conducted in compliance with all the regulations stated by the Institutional Animal Care and Use Committee (IACUC) of the University of Georgia (UGA; under AUP A2019 01 – 004-A2). Vaccination and challenge studies were conducted under ABSL-2 conditions at the Davison Life Sciences Complex, UGA. Animal studies and procedures were performed according to the Institutional Animal Care and Use Committee Guidebook of the Office of Laboratory Animal Welfare and PHS policy on Humane Care and Use of Laboratory Animals. Animal studies were carried out in compliance with the ARRIVE guidelines (https://arriveguidelines.org).

### Cells, eggs, and mice

Madin-Darby canine kidney (MDCK) and human embryonic kidney 293T cells (HEK293T) were a kind gift from Robert Webster, St Jude Children’s Research Hospital, Memphis, TN, USA. Cells were used for the rescue of the different viruses used in this study. Cells were maintained at 37 °C, 5% CO_2_, in Dulbecco’s modified Eagle’s media (DMEM) supplemented with 10% fetal bovine serum (ThermoFisher Scientific, Waltham, MA, USA) and 1X antibiotic/antimycotic solution (ATB/ATM, 100 IU/mL of penicillin, 100 µg/mL for streptomycin, and 0.25 µg/mL of amphotericin B, Gibco, ThermoFisher Scientific). Specific pathogen-free (9–11 days old) embryonated chicken eggs (ECEs) used for virus propagation and stock titration were obtained from Charles River (Wilmington, MA).

Male and female DBA/2J mice (5 weeks old) were purchased from Jackson Laboratories (Bar Harbor, ME) and raised until 7 weeks of age. Mice were housed in negative pressure caging in the Davison Life Sciences Complex, University of Georgia, and were provided food and water *ad libitum* for the duration of the experiment.

### Recombinant plasmids

The PB1 plasmids containing the re-arranged M (PB1BM2) and re-arranged NS (PB1BNS2) and the M and NS plasmids encoding early stops codons have been previously described^17^. The PB1 plasmid containing the temperature sensitive mutations and C-terminal HA tag (pDP-PB1att) was previously described^20,28^. The plasmid pDP_B/Bris-PB1_Nluc (B/Bris) was produced carrying a chimeric PB1 gene segment from B/Brisbane/60/2008 (B/Bris) WT with an in-frame C-terminal nanoluciferase ORF (Nluc) between the C-terminal end of the PB1 ORF and the 3’UTR. The plasmid pDP2018-FluB-IGIP-HA was produced by subcloning the ORF of the B/Bris BHA into a reverse genetics vector carrying a synthetic DNA fragment (Genscript, Piscataway, NJ, USA) encoding the 5′ untranslated region (UTR) and signal peptide sequence of BHA (B/Bris), IGIP mature peptide sequence (24aa), and sequences encoding a G4S linker, a furin cleavage site, the Thosea assigna virus (TAV) 2A protease, and the transmembrane domain, cytoplasmic tail, and 3’ UTR of BHA. To generate the plasmids pCAGGS-GFP-IGIP-His and pCAGGS-GFP, an EcoRI-BglII DNA fragment encoding the ORF of a chimeric protein of the enhanced green fluorescent protein (GFP), IGIP, and a 6x HIS tag, or the GFP ORF respectively were generated by Twist DNA Biosciences (San Francisco, CA) and cloned into pCAGGS previously digested with the same enzymes. Plasmids were propagated in E. coli Top10 cells (ThermoFisher Scientific). Plasmids were purified using QIAGEN Plasmid Maxi Kit (Qiagen, Gaithersburg, MD, USA). Plasmid sequences were confirmed by Whole Plasmid Sequencing (Psomagen, Rockville, MD, USA).

### Rescue of recombinant FluB-RAM/IGIP, FluB-RANS/IGIP, and Flu-att/IGIP viruses

Rescue of recombinant FluB-RAM, FluB-RANS, and Flu-att viruses were previously described^20,28,45^. Rescue of FluB-RAM/IGIP, FluB-RANS/IGIP, and FluB-att/IGIP was performed similarly. Briefly, plasmids encoding wild type gene segments from B/Bris (PB2, PA, NP, NA and either NS and/or M, respectively) were mixed with the corresponding set of plasmids to produce the FluB-RAM/IGIP (pSCG-PB1BM2, pDP2018-FluB-HA-IGIP and pSCG-BM1-∆M2), FluB-RANS/IGIP (pSCG-PB1BNS2, pDP2018-FluB-HA-IGIP and pSCG-BNS1-∆NEP), and FluB-att/IGIP (pDP-PB1att and pDP2018-FluB-HA-IGIP) viruses. Recombinant viruses were propagated and titrated in 11-day-old SPF ECEs incubated at 33 °C for 48 h. First passage in ECEs (E1) virus stocks were stored at −80 °C until further use.

### Stability of recombinant viruses through Serial Passages in ECEs

Six additional serial passages were performed in 11-day-old ECEs from the E1 stock until E7 as previously described^17^. Aliquots from each passage were stored at −80 °C until needed. RNA was extracted from allantoic fluids collected at each passage and from the original virus stock using the QIAmp Viral RNA isolation kit (QIAGEN). The PB1, HA, M, and/or NS gene segments were amplified by RT-PCR using SuperScript III One-Step RT-PCR System with Platinum Taq DNA Polymerase (ThermoFisher Scientific). Sanger sequencing (Psomagen) was then performed from the resulting RT-PCR products to assess the stability after passaging by confirming the rearrangement of the PB1 gene segments, the presence of the IGIP coding sequence, and the early stop mutations within either the M or the NS gene segments, respectively.

### Virus growth kinetics

MDCK cells were seeded in 6-well plates and incubated overnight at 37 °C, 5% CO_2_. The next day, cells were inoculated with 0.01 MOI of either B/Bris WT or recombinant viruses contained in 500 µL, each in triplicate wells. Inoculated MDCK cells were incubated for 1 h at 35 °C, 5% CO_2_, gently rocking the plates every 15 min. Next, the virus inoculum was removed, cells were washed twice with PBS and replenished with 2 mL of fresh Opti-MEM I (ThermoFisher Scientific) supplemented with 1X ATB/ATM and 1 µg/mL of L-1-tosylamide-2-phenylethyl chloromethyl ketone (TPCK)-treated Trypsin. Plates were incubated at either 33°, 35°, or 37 °C, 5% CO_2_. Supernatants (200 µL) were collected at 0, 12, 24, 48, 72, and 96 h post-inoculation (hpi) and stored at −80 °C until processed. Samples were titrated by TCID_50_ in MDCK cells. Virus titers were calculated using the Reed and Muench protocol and plotted as the mean TCID_50_/mL ±SD^46^.

### Western-blot analysis

MDCK cells were seeded in 6-well plates and incubated overnight at 37 °C, 5% CO_2_. The next day, cells were inoculated with 0.1 MOI of the indicated viruses. Inoculated MDCK cells were incubated for 1 h at 35 °C, 5% CO_2_, gently rocking the plates every 15 min. Next, the virus inoculum was removed, cells were washed twice with PBS and replenished with 2 mL of fresh Opti-MEM supplemented with 1X ATB/ATM and 1 µg/mL of TPCK-treated Trypsin. At 16 h post-infection, the tissue culture supernatant was removed, and cells were lysed with 1X RIPA buffer (25 mM Tris.HCl pH 7.6, 150 mM NaCl, 1% NP-40, 1% sodium deoxycholate, and 0.1% SDS; ThermoFisher Scientific) supplemented with 1X Halt Protease Inhibitor Cocktail (ThermoFisher Scientific). HEK293T cells were transfected with 10 µg of pCAGGS-GFP-IGIP-His, pCAGGS-GFP, or pCAGGS and at 24 h post transfection the cells were lysed as described above. Protein concentration was quantified using the Pierce BCA Protein Assay Kit (ThermoFisher Scientific). Protein lysates were standardized to a concentration of 0.4 µg/µL and diluted 1:2 in 2X Laemmli buffer supplemented with 5% b-mercaptoethanol and incubated for 5 min at 100 °C. Samples (2 µg of protein/sample) were loaded in a 12% Mini-PROTEAN TGX Stain-Free Protein Gels (Bio-Rad, Hercules, CA) and resolved at 100 V for 5 min, followed by 2 h at 175 V. Proteins were transferred to a 0.2 µm nitrocellulose membrane (Supplementary Figure 1) using the Trans-Blot Turbo Transfer System (Bio-Rad) at 2.5 amps for 7 min. After, the membrane was blocked overnight at 4 °C using H20-milk 5%. The next day, anti-GAPDH (Santa Cruz Biotechnology; 1:1000), anti-FLUBV-HA (Sino Biological; 1:5000), anti-FLUBV-NP (Invitrogen; 1:250), mouse polyclonal anti-FLUBV (1:500), anti-IGIP (Invitrogen; 1:500) were diluted in H20-BSA 3% and incubated with the membranes for 2 h at room temperature while shaking. The membranes were then washed 3 times with 1X PBS + 0.05% Tween 20 for 5 min each wash. After, the membranes were incubated with the respective secondary antibody diluted in H20-milk 5% for 1 h at room temperature while shaking. Anti-mouse IgG-horseradish peroxidase (HRP) conjugate (Invitrogen; 1:5000 for GADPH and polyclonal anti-FLUBV detection), anti-rabbit IgG-HRP conjugate (Abcam; 1:100000 for IGIP, FLUBV-HA and FLUBV-NP detection), or StrepTactin-HRP Conjugate (Bio-Rad; 1:10000 for molecular marker) were used. Finally, the membranes were washed 3 times with 1X PBS + 0.05% Tween 20 and imaged through a chemiluminescent reaction using West Pico (FLUBV-NP, FLUBV-HA, and GADPH; ThermoFisher) or West Femto (mouse polyclonal anti-FLUBV and IGIP; ThermoFisher). The ChemiDoc MP Imaging System (Bio-Rad) was employed to visualize the membranes and capture the images.

### Vaccine safety and efficacy

Please note that the vaccine safety and efficacy study described herein was part of a larger study that included a set of recombinant viruses whose efficacy profiles have been previously described^17,28^. Following a prime-boost strategy 20 days apart, 7-week-old DBA/2J mice were primed intranasally (i.n.) with 50 µL of inoculum at a virus dose of 10^6^ EID_50_/mouse. Male and female mice, housed separately, were allocated into 8 groups (½ males and ½ females) as follows: Mock-vaccinated (mock-vac, PBS, *n* = 24); FluB-RAM/IGIP (*n* = 12); FluB-RANS/IGIP (*n* = 12); FluB-att/IGIP (*n* = 12), FluB-RAM (*n* = 12); FluB-RANS (*n* = 12); FluB-att (*n* = 12); and B/Brisbane/60/2008 (B/Bris WT, *n* = 12, control virus). Mice were monitored daily for clinical signs, body weight changes, and mortality for up to 12 days following vaccination (dpv) and boost (dpb). On day 19 dpb, a subset of mice from each group (*n* = 4/group, ½ females, except where noted) was anesthetized with isoflurane, terminally bled to collect sera, and subsequently humanely euthanized (Fig. 2a, study design).

Mice from the vaccine safety study (*n* = 8/group, ½ females) were challenged i.n. with a lethal dose (10^7^ EID_50_/mouse, ~1000 MLD50) of the B/Brisbane/60/2008 PB2-F406Y (B/Bris/F406Y) strain^20^ contained in 50 µL of PBS. A subset of mice in the mock group (*n* = 8, ½ females) remained unchallenged and served as negative controls. Mice were monitored twice daily to record clinical signs and mortality for up to 14 days post-challenge (dpc). Bodyweight was recorded for up to 12 dpc. At 14 dpc, survivors were anesthetized with isoflurane, terminally bled to collect sera, and subsequently humanely euthanized (Fig. 2a, study design).

### Hemagglutination inhibition (HI) assay

RDE-treated sera collected at 19 dpb (*n* = 4/group, ½ females) and 14 dpc (*n* = 8/group, ½ females) was used for HI assays performed in V-bottom microtiter plates, using 4 hemagglutination units (HAU) of viral antigen (B/Bris WT) per 25 µL, as recommended by the OIE and as previously described^29^, using a suspension of turkey red blood cells (0.5%). HI titers were plotted using Prism v9 (GraphPad, San Diego, CA, USA). The limit of detection was a dilution of 1/10, and samples with undetectable titers were assigned a dilution value of 1/8 for statistical purposes.

### Recombinant B/Brisbane/60/2008 and B/Wisconsin/01/2010 viruses expressing nano luciferase (Nluc), B/Bris/Nluc and B/Wis/Nluc

The recombinant Victoria lineage B/Bris/Nluc and Yamagata lineage B/Wis/Nluc viruses expressing PB1-Nluc were generated for a modified virus neutralization assay as previously described^18^. Rescue of B/Bris/Nluc was achieved with 7 B/Bris WT plasmids co-transfected with pDP_ B/Bris-PB1_Nluc (7 + 1). Rescue of B/Wis/NLuc was performed using 5 plasmids from B/Bris WT co-transfected with pDP_ B/Bris-PB1_Nluc, and the plasmids encoding the HA and NA from B/Wisconsin/01/2010. The recombinant viruses were propagated in 11-day-old ECEs and titrated by TCID_50_ in MDCK cells. Virus identities were confirmed by Sanger sequencing. Nluc activity from infected cells was detected using the Nano-Glo Luciferase Assay System (Promega, Madison, WI) following manufacturer’s instructions.

### Virus Neutralization Assay based on Nluc activity (VNluc)

Two-fold dilutions in PBS of sera treated with receptor destroying enzyme (RDE; Denka Seiken, VWR, PA, USA) collected at 19 dpb (*n* = 8/group, ½ females) was used for VNluc assays. 100 TCID_50_ contained in 50 µL of either B/Bris/Nluc (homologous) or B/Wis/Nluc (heterologous) were added to each of the corresponding wells containing serum dilutions. Serum/virus mixes were incubated at 37 °C for 1 h. Thereafter, the serum/virus mixes were added to MDCK cell monolayers and set to incubate at 4 °C for 15 min and then at 35 °C for 45 min. After incubation, the serum/virus mixes were removed from the cell monolayers and 200 µL of Opti-MEM (Gibco) supplemented with 1X ATB/ATM and 1 µg/mL of TPCK-Trypsin was added to each well. Plates were set to incubate for 72 h at 35 °C, under 5% CO2. Virus neutralization titers were determined by measuring luminescence activity using the Nano-Glo Luciferase Assay System (Promega, Madison, WI) following the manufacturer’s instructions.

### Microarray for IgG and IgA determination

Sera collected at 19 dpb and 14 dpc, and nasal washes collected at 14 dpc were analyzed through protein microarrays to determine anti-HA, -NA, and -NP IgG and IgA levels from multiple Victoria- and Yamagata-like FLUBVs (Table 1). Purified FLUBV protein antigens were purchased from Sino Biological (Wayne, PA) (Table 1). Microarrays were carried out as described previously^18,47^. The results are expressed as the group mean fluorescence intensity (MFI) ± SD. The higher the MFI, the more Abs bound to a particular antigen. Due to low MFI signals, those from HA1 B/Florida/4/2006 (Catalog# 11053-V08H1, HEK293), HA1 B/Brisbane/60/2008 (Catalog# 40016-V08B, E. coli), and NA B/Brisbane/60/2008 (Catalog# 40203-VNAHC, HEK293) were not utilized in the analyses. MFIs were plotted and analyzed using Prism v9 (GraphPad).

### Statistical analyses

Virus growth kinetics were analyzed using the Gompertz growth non-linear regression model followed by Area Under the Curve (AUC) analysis, and Brown-Forsythe and Welch ANOVA plus Dunnett’s T3 post hock analysis to identify differences in growth rate between vaccine candidates. Two-way ANOVA was employed to determine virus growth differences by timepoint. VNluc assay curves were analyzed using multiple t-tests followed by the Holm-Sidak method to correct for multiple comparisons. Pre-challenge and post-challenge mean HI titers were analyzed by 2-way ANOVA followed by post hock Sidak’s multiple comparison test to determine differences between sex and vaccine treatment groups. Survival curves were analyzed using the Log-rank test. Brown–Forsythe and Welch ANOVA or Two-way ANOVA were performed to analyze the microarray data to compare responses between sex and/or vaccine groups, followed by a Dunnett’s T3 or a Tukey’s multiple comparisons test, respectively. The level of significance for all the analysis was considered at *p* < 0.05. *P* values were adjusted to account for multiple comparisons. All the analyses were performed using Prism v9.3.1.

### Reporting summary

Further information on research design is available in the Nature Research Reporting Summary linked to this article.

### Supplementary information

Supplementary Figures

REPORTING SUMMARY

## Data Availability

The protein microarray data discussed in this publication have been deposited in NCBI’s Gene Expression Omnibus^48^ and are accessible through GEO Series accession number GSE180642 and GSE205172.
