# Supplementary material for: Impact of sex on humoral immunity with live influenza B virus vaccines in mice
Source: NPJ Vaccines. 2024 Feb 26;9:45. doi: 10.1038/s41541-024-00827-x (PMC10897209; doi:10.1038/s41541-024-00827-x)
Supplement: Supplementary file 1 — Supplementary Figures [file 41541_2024_827_MOESM1_ESM.pdf]

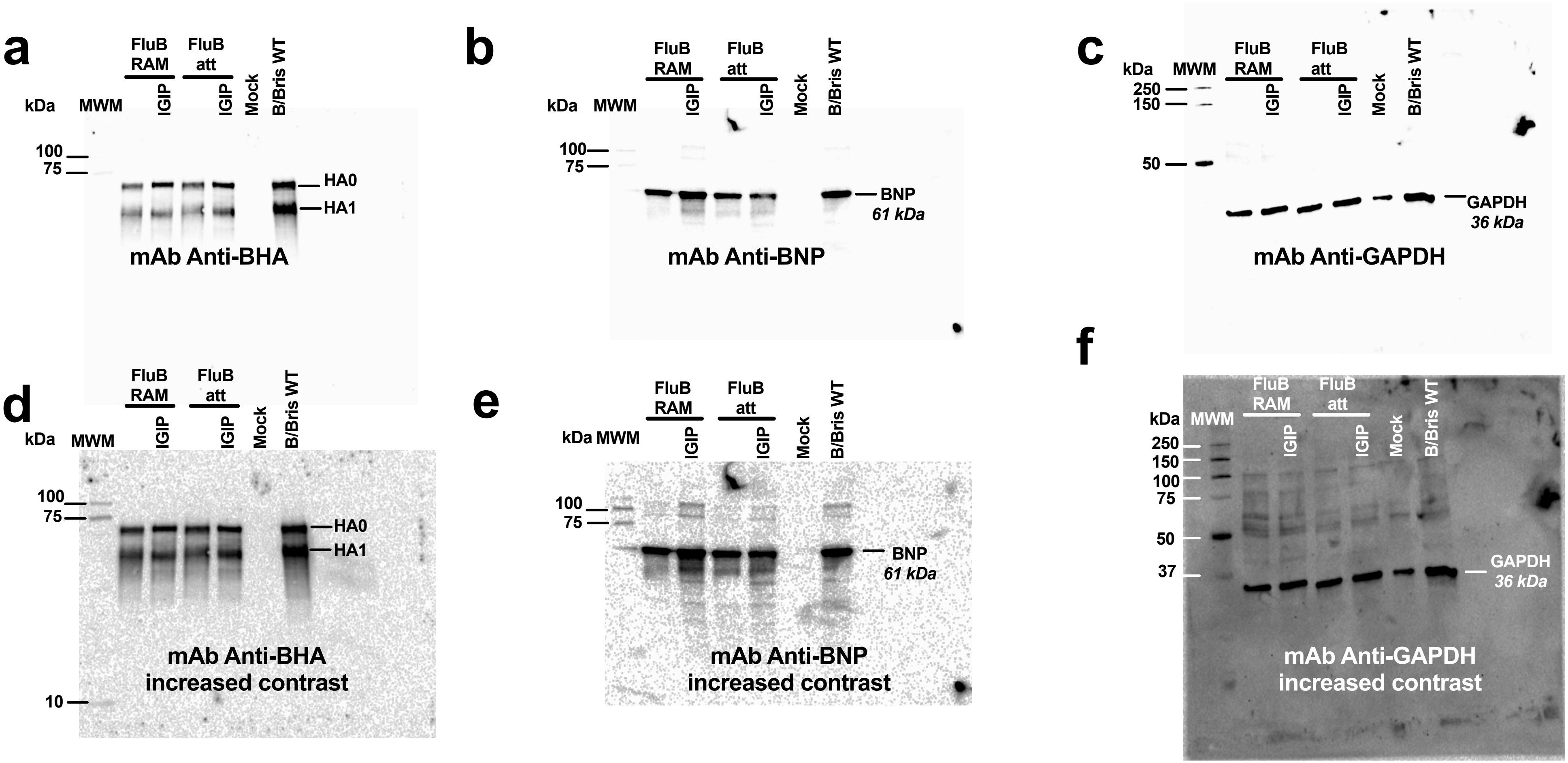

Supplementary Figure 1. Western blot analyses showing the entire nitrocellulose membranes probed with different antibodies as described in Figure 1D. (a-f) MDCK cells were seeded in 6-well plates. The next day, cells were inoculated with 0.1 MOI of the indicated viruses. At 16 h post-infection, the tissue culture supernatant was removed, and cells were lysed with 1X RIPA buffer supplemented with 1X Halt Protease Inhibitor Cocktail. Protein lysates from recombinant or WT FLUBV infected cells and control non-infected cells probed with **(a, d)** anti-BHA antibody, **(b, e)** anti-BNP, or **(c, f)** anti-GADPH (control). The contrast of membranes a, b, and c was increased to generate d, e, and f, respectively. Protein concentration was quantified using the Pierce BCA Protein Assay Kit. Protein lysates were standardized to a concentration of 0.4 µg/uL and diluted 1:2 in 2X Laemmli buffer supplemented with 5% b-mercaptoethanol and incubated for 5 min at 100°C. Samples (2 µg of protein/sample) were loaded in a 12% Mini-PROTEAN TGX Stain-Free Protein Gels (Bio-Rad, Hercules, CA) and resolved at 100V for 5 min, followed by 2 h at 175V. Proteins were transferred to a 0.2 µm nitrocellulose membrane using the Trans-Blot Turbo Transfer System (Bio-Rad) at 2.5 amps for 7 min. After, the membrane was blocked overnight at 4°C using H20-milk 5%. The next day, anti-GAPDH (Santa Cruz Biotechnology; 1:1000), anti-FLUBV-HA (Sino Biological; 1:5000), anti-FLUBV-NP (Invitrogen; 1:250), were diluted in H20-BSA 3% and incubated with the membranes for 2 h at room temperature while shaking. The membranes were then washed 3 times with 1X PBS + 0.05% Tween 20 for 5 min each wash. After, the membranes were incubated with the respective secondary antibody diluted in H20-milk 5% for 1 h at room temperature while shaking. Anti-mouse IgG-horseradish peroxidase (HRP) conjugate (Invitrogen; 1:5000 for GADPH), anti-rabbit IgG-HRP conjugate (FLUBV-HA and FLUBV-NP detection), or StrepTactin-HRP Conjugate (Bio-Rad; 1:10000 for molecular marker) were used. Finally, the membranes were imaged through a chemiluminescent reaction. The ChemiDoc MP Imaging System (Bio-Rad) was employed to visualize the membranes, capture and produce images with different contrasts.

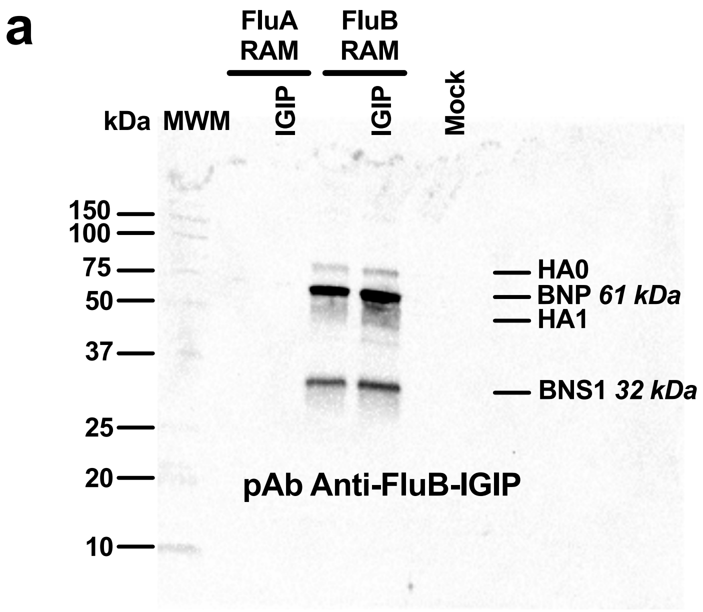

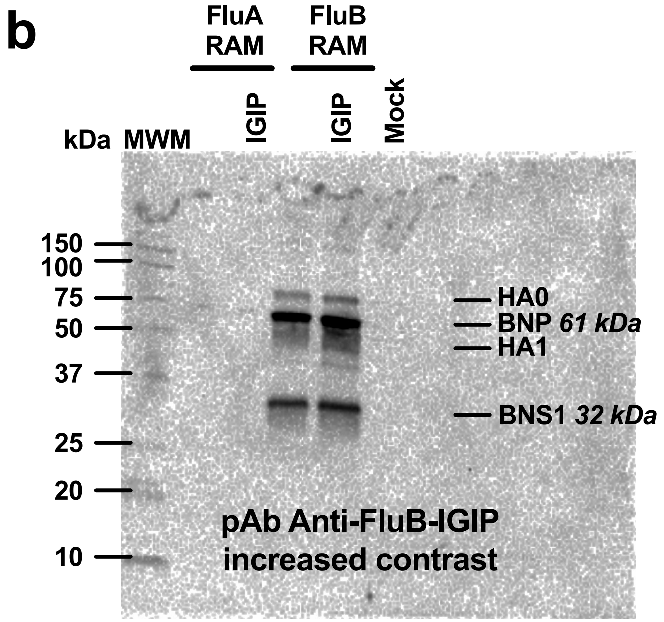

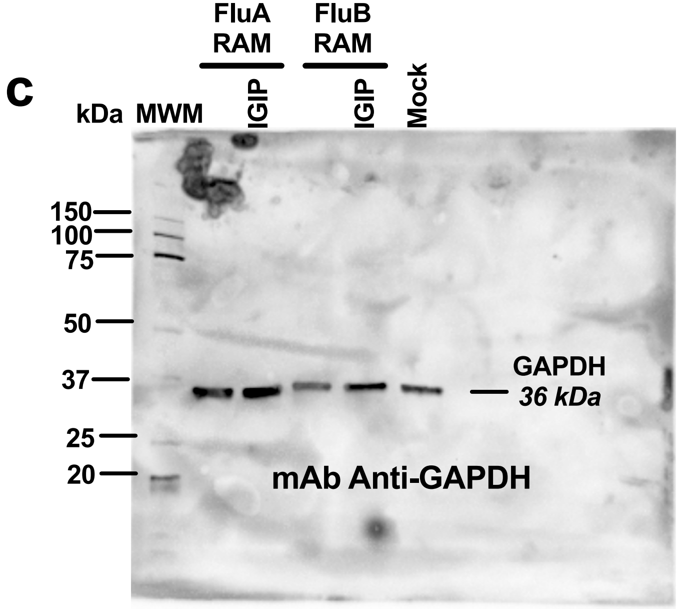

Supplementary Figure 2. Western blot analyses showing the entire nitrocellulose membranes probed with different antibodies as described in Figure 1E. (a-d) MDCK cells were seeded in 6-well plates. The next day, cells were inoculated with 0.1 MOI of the indicated viruses. At 16 h post-infection, the tissue culture supernatant was removed, and cells were lysed with 1X RIPA buffer supplemented with 1X Halt Protease Inhibitor Cocktail. Protein lysates from recombinant viruses FLUA-RAM (with or without IGIP), FLUB-RAM (with or without IGIP) infected cells and control non-infected cells probed with (a) Polyclonal sera anti-FluB-RAM/IGIP or (c) anti-GADPH (control). The contrast of membrane a) was increased to generate b). Protein concentration was quantified using the Pierce BCA Protein Assay Kit. Protein lysates were standardized to a concentration of 0.4 µg/uL and diluted 1:2 in 2X Laemmli buffer supplemented with 5% b-mercaptoethanol and incubated for 5 min at 100°C. Samples (2 µg of protein/sample) were loaded in a 12% Mini-PROTEAN TGX Stain-Free Protein Gels (Bio-Rad, Hercules, CA) and resolved at 100V for 5 min, followed by 2 h at 175V. Proteins were transferred to a 0.2 µm nitrocellulose membrane using the Trans-Blot Turbo Transfer System (Bio-Rad) at 2.5 amps for 7 min. After, the membrane was blocked overnight at 4°C using H20-milk 5%. The next day, anti-GAPDH (Santa Cruz Biotechnology; 1:1000) and mouse polyclonal anti-FLUBV (1:500) were diluted in H20-BSA 3% and incubated with the membranes for 2 h at room temperature while shaking. The membranes were then washed 3 times with 1X PBS + 0.05% Tween 20 for 5 min each wash. After, the membranes were incubated with the respective secondary antibody diluted in H20-milk 5% for 1 h at room temperature while shaking. Anti-mouse IgG-horseradish peroxidase (HRP) conjugate (Invitrogen; 1:5000 for GADPH and polyclonal anti-FLUBV detection) or StrepTactin-HRP Conjugate (Bio-Rad; 1:10000 for molecular marker) were used. Finally, the membranes were imaged through a chemiluminescent reaction. The ChemiDoc MP Imaging System (Bio-Rad) was employed to visualize the membranes and capture and contrast the images.

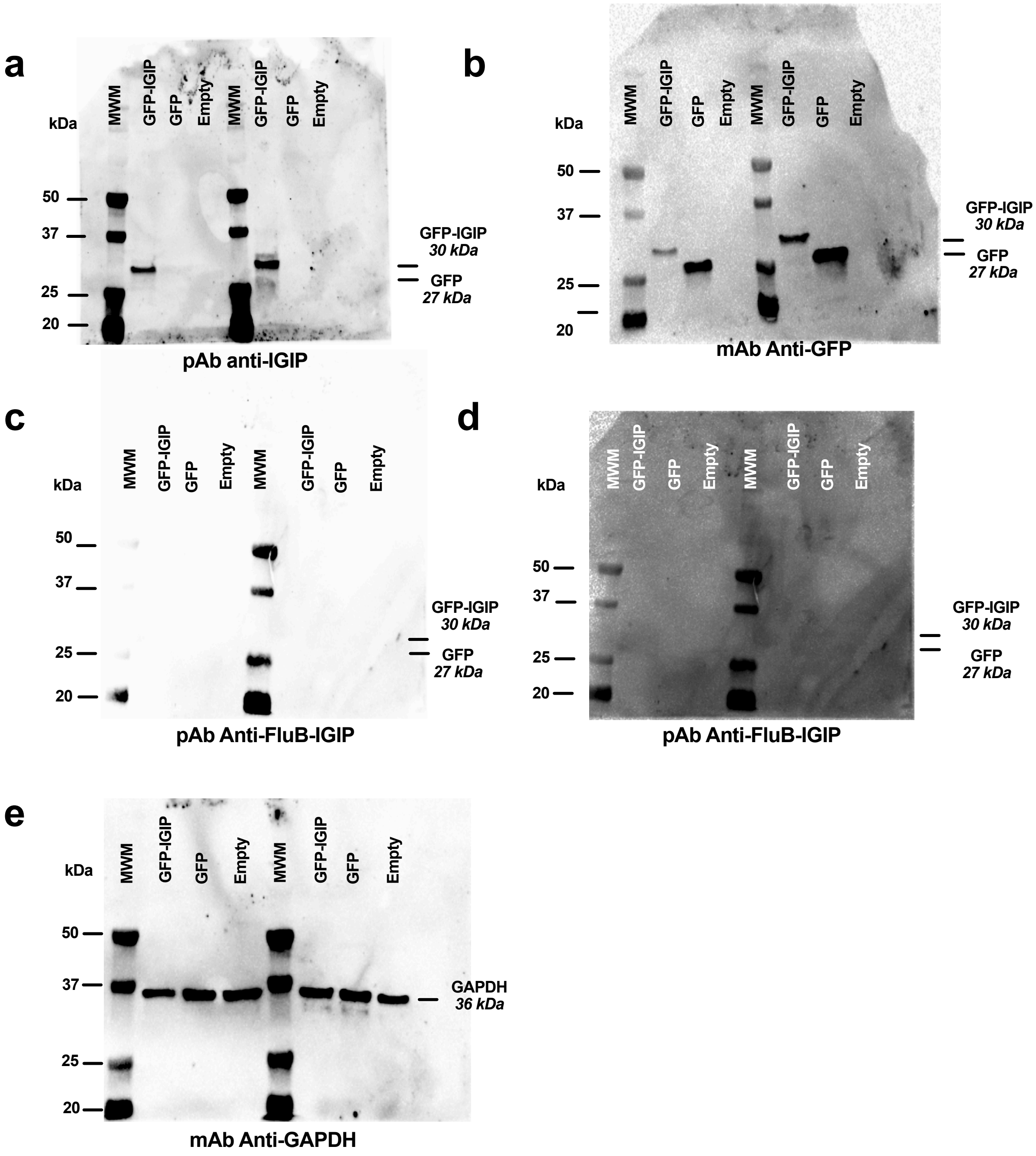

Supplementary Figure 3. Western blot analyses showing the entire nitrocellulose membranes probed with different antibodies as described in Figure 1F. (a-e) HEK293T cells were transfected with 10 µg of pCAGGS-GFP-IGIP-His, pCAGGS-GFP, or pCAGGS. Twenty-four hours post-transfection, the cells were lysed with 1X RIPA buffer supplemented with 1X Halt Protease Inhibitor Cocktail. Protein lysates from transfected cells and control cells were probed with **(a)** anti-IGIP, **(b)** anti-GFP, **(c-d)** Polyclonal sera anti-FluB-RAM/IGIP, or **(e)** anti-GADPH. The contrast of membrane c) was increased to generate d). Protein concentration was quantified using the Pierce BCA Protein Assay Kit. Protein lysates were standardized to a concentration of 0.4 µg/uL and diluted 1:2 in 2X Laemmli buffer supplemented with 5% b-mercaptoethanol and incubated for 5 min at 100°C. Samples (2 µg of protein/sample) were loaded in duplicates a 12% Mini-PROTEAN TGX Stain-Free Protein Gels (Bio-Rad, Hercules, CA) and resolved at 100V for 5 min, followed by 2 h at 175V. Proteins were transferred to a 0.2 µm nitrocellulose membrane using the Trans-Blot Turbo Transfer System (Bio-Rad) at 2.5 amps for 7 min. After, the membrane was blocked overnight at 4°C using H20-milk 5%. The next day, anti-GAPDH (Santa Cruz Biotechnology; 1:1000), anti-IGIP (Invitrogen; 1:500), anti-GFP (Abcam; 1:1000), and mouse polyclonal anti-FLUBV (1:500) were diluted in H20-BSA 3% and incubated with the membranes for 2 h at room temperature while shaking. The membranes were then washed 3 times with 1X PBS + 0.05% Tween 20 for 5 min each wash. After, the membranes were incubated with the respective secondary antibody diluted in H20-milk 5% for 1 h at room temperature while shaking. Anti-mouse IgG-horseradish peroxidase (HRP) conjugate (Invitrogen; 1:5000 for GADPH and polyclonal anti-FLUBV detection), anti-rabbit IgG-HRP conjugate (IGIP and GFP detection), or StrepTactin-HRP Conjugate (Bio-Rad; 1:10000 for molecular marker) were used. Finally, the membranes were imaged through a chemiluminescent reaction. The ChemiDoc MP Imaging System (Bio-Rad) was employed to visualize the membranes and capture the images.
