# Supplementary material for: Impact of sex on humoral immunity with live influenza B virus vaccines in mice
Source: NPJ Vaccines. 2024 Feb 26;9:45. doi: 10.1038/s41541-024-00827-x (PMC10897209; doi:10.1038/s41541-024-00827-x)
Supplement: Supplementary file 2 — REPORTING SUMMARY [file 41541_2024_827_MOESM2_ESM.pdf]

nature portfolio

Corresponding author(s): Perez, Daniel R.

Last updated by author(s): Feb 2, 2024

Reporting Summary

Nature Portfolio wishes to improve the reproducibility of the work that we publish. This form provides structure for consistency and transparency in reporting. For further information on Nature Portfolio policies, see our Editorial Policies and the Editorial Policy Checklist.

Statistics

For all statistical analyses, confirm that the following items are present in the figure legend, table legend, main text, or Methods section.

n/a	Confirmed	
☐	☒	The exact sample size (*n*) for each experimental group/condition, given as a discrete number and unit of measurement
☐	☒	A statement on whether measurements were taken from distinct samples or whether the same sample was measured repeatedly
☐	☒	The statistical test(s) used AND whether they are one- or two-sided<br>*Only common tests should be described solely by name; describe more complex techniques in the Methods section.*
☒	☐	A description of all covariates tested
☒	☐	A description of any assumptions or corrections, such as tests of normality and adjustment for multiple comparisons
☐	☒	A full description of the statistical parameters including central tendency (e.g. means) or other basic estimates (e.g. regression coefficient) AND variation (e.g. standard deviation) or associated estimates of uncertainty (e.g. confidence intervals)
☒	☐	For null hypothesis testing, the test statistic (e.g. *F*, *t*, *r*) with confidence intervals, effect sizes, degrees of freedom and *P* value noted<br>*Give P values as exact values whenever suitable.*
☒	☐	For Bayesian analysis, information on the choice of priors and Markov chain Monte Carlo settings
☒	☐	For hierarchical and complex designs, identification of the appropriate level for tests and full reporting of outcomes
☒	☐	Estimates of effect sizes (e.g. Cohen's *d*, Pearson's *r*), indicating how they were calculated

Our web collection on statistics for biologists contains articles on many of the points above.

Software and code

Policy information about availability of computer code

Data collection	Data collection was adquired and stored using microsoft excel. Clinical signs were recorded in a in house developed form and subsequentenly transferred to Microsoft excel.
Data analysis	All data analysis was performed using Prism v9.3.1.

For manuscripts utilizing custom algorithms or software that are central to the research but not yet described in published literature, software must be made available to editors and reviewers. We strongly encourage code deposition in a community repository (e.g. GitHub). See the Nature Portfolio guidelines for submitting code & software for further information.

Data

Policy information about availability of data

All manuscripts must include a data availability statement. This statement should provide the following information, where applicable:

- Accession codes, unique identifiers, or web links for publicly available datasets
- A description of any restrictions on data availability
- For clinical datasets or third party data, please ensure that the statement adheres to our policy

The protein microarray data discussed in this publication have been deposited in NCBI’s Gene Expression Omnibus 48 and are accessible through GEO Series accession number GSE180642 and GSE205172.

Research involving human participants, their data, or biological material

Policy information about studies with human participants or human data. See also policy information about sex, gender (identity/presentation), and sexual orientation and race, ethnicity and racism.

Reporting on sex and gender	Not applicable
Reporting on race, ethnicity, or other socially relevant groupings	Not applicable
Population characteristics	Not applicable
Recruitment	Not applicable
Ethics oversight	Not applicable

Note that full information on the approval of the study protocol must also be provided in the manuscript.

Field-specific reporting

Please select the one below that is the best fit for your research. If you are not sure, read the appropriate sections before making your selection.

☒ Life sciences ☐ Behavioural & social sciences ☐ Ecological, evolutionary & environmental sciences

For a reference copy of the document with all sections, see nature.com/documents/nr-reporting-summary-flat.pdf

Life sciences study design

All studies must disclose on these points even when the disclosure is negative.

Sample size	No sample size calculation was performed. Number of animals/group (n=12) meets and exceeds the standards for these types of studies in the field.
Data exclusions	No data was excluded in the analyses.
Replication	Virus growth kinetics were analyzed using the Gompertz growth non-linear regression model. Differences in growth rate between viruses were calculated by AUC analysis, followed by Brown-Forsythe and Welch ANOVA plus Dunnett's T3 post hock analysis. Two-way ANOVA was employed to determine virus growth differences by timepoint. Statistically significant differences between viruses' growth rates are denoted. Western blot analyses were repeated at least twice starting from repeated infections/transfections.
Randomization	Mice were randomly assigned to different group. Dfferent analyses were performed based on vaccine backbone, presence/absence of IGIP, and impact of biological sex as extensively detailed in the manuscript.
Blinding	The study design, particularly during the animal study, required that personnel be fully aware of the groups in question to avoid errors in the allocation of data points during data collection. Personnel working and generating protein microarray data were blind to sample type and group allocation.

Reporting for specific materials, systems and methods

We require information from authors about some types of materials, experimental systems and methods used in many studies. Here, indicate whether each material, system or method listed is relevant to your study. If you are not sure if a list item applies to your research, read the appropriate section before selecting a response.

Materials & experimental systems

n/a	Involved in the study	
☐	☒	Antibodies
☐	☒	Eukaryotic cell lines
☒	☐	Palaeontology and archaeology
☐	☒	Animals and other organisms
☒	☐	Clinical data
☒	☐	Dual use research of concern
☒	☐	Plants

Methods

n/a	Involved in the study	
☒	☐	ChIP-seq
☒	☐	Flow cytometry
☒	☐	MRI-based neuroimaging

Antibodies

Antibodies used	The following primary antibodies were used in this study: 1) Influenza B (B/Massachusetts/03/2010) Hemagglutinin / HA1 Antibody, Rabbit PAb, Antigen Affinity Purified, Sino Biological, catalog number #40191-T56. 2) Influenza B NP (B/Florida/4/2006) Polyclonal Antibody, Invitrogen, catalog number #PA5-81758, lot number XE3571622A. 3) IGIP Polyclonal Antibody, Invitrogen, catalog number #PA5-65316, lot number YD3882996. 4) GAPDH Monoclonal Antibody (6C5), Invitrogen, catalog number #AM4300, lot number 2673398. 5) Recombinant Anti-GFP antibody [EPR14104], abcam, catalog number #ab183734, lot number 1033451-2. 5) Polyclonal anti-influenza B raised in mice, developed in house. The following seconday antibodies were used:1) Goat anti-Mouse IgG (H+L) Secondary Antibody, HRP, invitrogen, catalog number #31430, lot number YD375335. 2) Goat Anti-Rabbit IgG H&L (HRP), abcam, catalog number #ab6721, lot number GR3251481-1. The molecular marker was detected using Precision Protein™ StrepTactin-HRP Conjugate, BioRad, catalog number #1610380, lot number 64333457
Validation	Influenza B (B/Massachusetts/03/2010) Hemagglutinin / HA1 Antibody, Rabbit PAb, Antigen Affinity Purified, Sino Biological, catalog number #40191 has been valudated for western blot (1:1000-1:5000) and ELISA (1:5000-1:10000). Influenza B NP (B/Florida/4/2006) Polyclonal Antibody, Invitrogen, catalog number #PA5-81758 has been validated for Western blot (1:1000-1:5000) and ELISA (1:5000-1:10000). IGIP Polyclonal Antibody, Invitrogen, catalog number #PA5-65316 has been validated for ICC/IFF (0.25ug/mL-2ug/mL). Validation for western blot was performed in house. Polyclonal anti-influenza B raised in mice, developed in house was validated for western blot in house. GAPDH Monoclonal Antibody (6C5), Invitrogen, catalog number #AM4300 has been validated for western blot (3ug/mL), IHC, ICC/IF (1ug/mL), IP and dot blot. Recombinant Anti-GFP antibody [EPR14104], abcam, catalog number #ab183734 has been validated for Flow citometry, western blot, IHC-P and ICC/IF.

Eukaryotic cell lines

Policy information about cell lines and Sex and Gender in Research

Cell line source(s)	Madin-Darby canine kidney (MDCK) and human embryonic kidney 293T cells (HEK293T) were a kind gift from Robert Webster, St Jude Children's Research Hospital, Memphis, TN, USA.
Authentication	Cell lines were not authenticated
Mycoplasma contamination	Mycoplasma testing is performed routinely in the lab using Fisher Scientific Universal Mycoplasma Detection Kit (catalog # 50189644FP)
Commonly misidentified lines (See ICLAC register)	Not applicable.

Animals and other research organisms

Policy information about studies involving animals; ARRIVE guidelines recommended for reporting animal research, and Sex and Gender in Research

Laboratory animals	Male and female DBA/2J mice (Strain #000671) were purchased from The Jackson Laboratory (Bar Harbor, ME) at 5 weeks old and raised until 7 weeks of age. DBA/2J mice are also known as DBA2, D2J, D2, DBA or DBA/2
Wild animals	Not applicable
Reporting on sex	Biological sex as a variable of immune responses is at the core of the manuscript. It was taken into account and reported throughout the manuscript.
Field-collected samples	Not applicable
Ethics oversight	Animal studies were approved and conducted in compliance with all the regulations stated by the Institutional Animal Care and Use Committee (IACUC) of the University of Georgia (UGA; under AUP A2019 01-004-A2). Vaccination and challenge studies were conducted under ABSL-2 conditions at the Davison Life Sciences Complex, UGA. Animal studies and procedures were performed according to the Institutional Animal Care and Use Committee Guidebook of the Office of Laboratory Animal Welfare and PHS policy on Humane Care and Use of Laboratory Animals. Animal studies were carried out in compliance with the ARRIVE guidelines (https://arriveguidelines.org).

Note that full information on the approval of the study protocol must also be provided in the manuscript.

Plants

Seed stocks	Not applicable
Novel plant genotypes	Not applicable
Authentication	Not applicable
